# Residual periodontal ligament in the extraction socket promotes the dentin regeneration potential of DPSCs in the rabbit jaw

**DOI:** 10.1186/s13287-023-03283-x

**Published:** 2023-03-20

**Authors:** Bin Luo, Yu Luo, Lin He, Yangyang Cao, Qingsong Jiang

**Affiliations:** 1 Department of Prosthodontics, Beijing Stomatological Hospital, School of Stomatology, Capital Medical University, Beijing, 100050 China; 2 Beijing Key Laboratory of Tooth Regeneration and Function Reconstruction, Beijing Stomatological Hospital, School of Stomatology, Capital Medical University, Beijing, 100050 China

**Keywords:** Residual periodontal ligament, Periodontal ligament stem cells, Dental pulp stem cells, Dentin regeneration

## Abstract

**Background:**

Because of the low regeneration efficiency and unclear underlying molecular mechanism, tooth regeneration applications are limited. In this study, we explored the influence of residual periodontal ligament on the dentin regeneration potential of dental pulp stem cells (DPSCs) in the jaw.

**Methods:**

To establish a tooth regeneration model, the incisors of New Zealand white rabbits were extracted while preserving residual periodontal ligament, followed by the implantation of DPSCs. After 3 months, micro-computed tomography (micro-CT), stereomicroscopy and scanning electron microscopy (SEM) were used to observe the volume, morphology and microstructure of regenerated tissue. Histological staining and immunostaining analyses were used to observe the morphological characteristics and expression of the dentin-specific proteins DMP1 and DSPP. To explore the mechanism, DPSCs and periodontal ligament stem cells (PDLSCs) were cocultured in vitro, and RNA was collected from the DPSCs for RNA-seq and bioinformatic analysis.

**Results:**

The results of micro-CT and stereomicroscopy showed that the number of sites with regeneration and the volume of regenerated tissue in the DPSCs/PDL group (6/8, 1.07 ± 0.93 cm^3^) were larger than those in the DPSCs group (3/8, 0.23 ± 0.41 cm^3^). The results of SEM showed that the regenerated dentin-like tissue in the DPSCs and DPSCs/PDL groups contained dentin tubules. Haematoxylin and eosin staining and immunohistochemical staining indicated that compared with the DPSCs group, the DPSCs/PDL group showed more regular regenerated tissue and higher expression levels of the dentin-specific proteins DMP1 and DSPP (DMP1: *P* = 0.02, DSPP: *P* = 0.01). RNA-seq showed that the coculture of DPSCs with PDLSCs resulted in the DPSCs differentially expressing 427 mRNAs (285 upregulated and 142 downregulated), 41 lncRNAs (26 upregulated and 15 downregulated), 411 circRNAs (224 upregulated and 187 downregulated), and 19 miRNAs (13 upregulated and 5 downregulated). Bioinformatic analysis revealed related Gene Ontology function and signalling pathways, including extracellular matrix (ECM), tumour necrosis factor (TNF) signalling and chemokine signalling pathways.

**Conclusions:**

Residual periodontal ligament in the extraction socket promotes the dentin regeneration potential of DPSCs in the jaw. RNA-seq and bioinformatic analysis revealed that ECM, TNF signalling and chemokine signalling pathways may represent the key factors and signalling pathways.

**Supplementary Information:**

The online version contains supplementary material available at 10.1186/s13287-023-03283-x.

## Background

Tooth loss caused by caries, periodontal disease, and trauma is a common oral condition. Some progress has been made in the prevention and treatment of oral diseases, and the structure and function of missing teeth can be restored in many ways; however, tooth loss remains an outstanding public health issue [1, 2]. Methods for replacing missing teeth include the use of traditional fixed bridges, removable partial dentures and implant restoration [3]. However, traditional restorations lack the periodontal ligament structure and cannot provide the same perception or stress buffering capability as natural teeth [4, 5]. With the development of stem cell biology and tissue engineering technology, researchers have attempted to regenerate tooth structures and even whole teeth [6, 7]. Regenerated teeth have biomechanical properties and periodontal ligament and dentin matrix structures similar to those of natural teeth [8–10]. Tooth regeneration strategies can be roughly divided into two categories. One type is based on an epithelial-mesenchymal bioengineered tooth germ, but this type of method is difficult to apply due to the limited cell resource and uncontrollable morphology of the regenerated teeth [11]. In contrast, another type of biological root regeneration strategy is based on the combination of mesenchymal stem cells (MSCs) and scaffold materials; this type of method is more feasible, and the combination of a preformed root scaffold and MSCs results in the formation of a functional tooth root in the alveolar bone [8, 9].

The ultimate goal of tooth regeneration research is to regenerate tooth structures similar to those of natural teeth and thus restore the missing teeth. Generally, the method for tooth regeneration in vivo is to prepare a cavity in the jawbone and then implant a composite consisting of a scaffold and MSCs. In a recent study, functional tooth roots were regenerated by implanting a composite comprising a root scaffold material and dental pulp stem cells (DPSCs) in the swine jawbone; however, compared with the 100% success rate of implant-supported dentures, the 21.7% success rate of regenerated biological tooth roots indicates low regeneration efficiency [10]. In these studies, an artificially prepared cavity was used as the implant bed for MSCs, and the bone-derived microenvironment in the cavity is more likely to induce the osteogenic differentiation of MSCs, resulting in low tooth regeneration efficiency in vivo. However, some studies have found that adding bone morphogenetic protein 2 (BMP2), secreted frizzled-related protein 2 (SFRP2) and other factors to the MSC microenvironment can improve the efficiency of dentin regeneration [12, 13], which indicates that regulation of the microenvironment may have a positive impact on the dentinogenic differentiation of MSCs.

A large number of studies have confirmed that the microenvironment has a very large impact on the physiological functions, pathological changes and therapeutic effects of stem cells [14, 15]. Physiologically, the microenvironment of MSCs is composed of various tissue components, cell populations and soluble factors, which strictly regulate the behaviour of MSCs [16, 17]. Under pathological conditions such as osteoporosis and periodontitis, the viability and differentiation of MSCs are severely impaired, leading to aggravation of the disease and impaired tissue healing [18–20]. In addition, in cell therapy and tissue engineering, the donor and recipient microenvironments play key roles in determining the regenerative efficacy of the transplanted MSCs [21, 22]. The extracellular matrix (ECM) is an important component of the cellular microenvironment. ECM obtained by decellularization contains a large number of growth factors and can significantly promote the proliferation and differentiation of MSCs [23]. In summary, these studies further demonstrate the key role of cell–microenvironment interactions in MSC-mediated tooth regeneration, and it is necessary to explore the influence of different microenvironments on MSC-mediated tooth regeneration.

Periodontal ligament (PDL) is a natural connective tissue between teeth and alveolar bone. Periodontal ligament is mainly composed of periodontal ligament fibres, cells and ECM [24, 25]. In the clinic, a large number of teeth are removed for different reasons. After a tooth is extracted, some periodontal ligament will remain in the extraction socket and maintain an odontogenic microenvironment in the extraction socket, different from the bone-derived microenvironment created by artificial cavity preparation. To clarify whether the odontogenic microenvironment maintained by residual periodontal ligament in the extraction socket can promote the dentin regeneration capability of MSCs, in this study, we extracted rabbit incisors while retaining the periodontal ligament structure in the fresh extraction socket to establish an odontogenic microenvironment and then implanted DPSCs. We explored the effect of the residual periodontal ligament microenvironment on the dentin regeneration potential of DPSCs in the rabbit jaw. The null hypothesis was that residual periodontal ligament in the extraction socket promotes the dentin regeneration potential of DPSCs in the rabbit jaw.

## Methods

New Zealand white rabbits were selected as experimental animals. The right upper and lower incisors were extracted, the residual periodontal ligament in the extraction socket was retained, and DPSCs were implanted to establish a tooth regeneration model. The dentin regeneration potential of the DPSCs in the residual periodontal ligament microenvironment was detected by micro-computed tomography (micro-CT), stereomicroscopy, scanning electron microscopy (SEM), histological staining and immunostaining. To explore the underlying mechanism, we cocultured rabbit DPSCs and periodontal ligament stem cells (PDLSCs) in vitro and collected DPSCs for RNA-seq and bioinformatic analysi**s.**

### Cell culture and identification

The animal research involved in this work was approved by the Animal Ethics and Walfare Committee of Beijing Stomatological Hospital Affiliated to Capital Medical University (Reference number: KQYY-202101-003 and KQYY-202111-005). The care and use of animals were performed strictly following the regulations on the management of experimental animals. Rabbit DPSCs and PDLSCs were obtained as previously described [26, 27]. Incisors were extracted from 3-month-old New Zealand white rabbits after oral disinfection. Phosphate-buffered saline (PBS) was used to rinse the tooth tissue and then collected the pulp and periodontal ligament. The pulp and periodontal ligament were cut with scissors and digested in 3 mg/ml type I collagenase and 4 mg/ml dispase for 1 h. After centrifugation, the rabbit DPSCs and PDLSCs were resuspended in Dulbecco's modified Eagle's medium containing foetal bovine serum and cultured in a cell incubator. The rabbit DPSCs and PDLSCs from passage 3–5 were used for subsequent research.

Flow cytometry was used to identify the surface markers of rabbit DPSCs before application. When the cell cultures reached 80–90% confluence, a cell suspension was obtained by trypsin digestion and aliquoted into a few sterile tubes, with each tube containing 1 × 10^6^ cells. Then, 5 µl (1:200) of anti- rabbit CD90 (cat no. ab225; Abcam, Cambridge, UK), CD105 (cat no. ab11414; Abcam), CD34 (cat no. ab81289; Abcam), CD45 (cat no. ab10558; Abcam), CD44 (cat no. MA5-28376, Invitrogen), and vimentin (cat no. GTX79851, GeneTex) antibodies were added to the samples and incubated at 4 °C for 60 min in the dark. After washing 3 times with PBS, the samples were incubated with the secondary antibody at 4 °C for 60 min, and then flow cytometry was used to identify the surface markers.

### Cell transplantation into the rabbit jaw

Twelve 3-month-old New Zealand white rabbits were randomly divided into three groups, i.e., the blank control group, the DPSCs group and the DPSCs/PDL group, with 4 rabbits in each group. All surgical procedures were completed under anaesthesia established with an intramuscular injection of 0.25 ml/kg Zoletil 50. After disinfecting the oral cavity, the right upper and lower incisors of the rabbit were removed. In the blank control group and the DPSCs group, each extraction socket was thoroughly cleaned to remove the residual periodontal ligament. In the DPSCs/PDL group, the residual periodontal ligament was retained in the extraction socket. In the blank control group, 150 µl of hydrogel was implanted, while in the DPSCs and DPSCs/PDL groups, a mixture of 100 µl of DPSCs suspension (1 × 10^6^ cells) and 50 µl of hydrogel was implanted. Absorbable sutures were then used to close the wound. Penicillin was injected intramuscularly for 3 days to avoid infection.

### Micro-CT, stereomicroscopy and SEM observation

Three months after the model was established, the rabbits were sacrificed, and the upper and lower jawbones were obtained for examination by micro-CT (80 kV, 2 s, Siemens Inveon, Munich, Germany). CTAn software was used to reconstruct and calculate the volume of regenerated tissue in the blank control, DPSCs and DPSCs/PDL groups.

After rabbit jawbones from the blank control, DPSCs and DPSCs/PDL groups were cut on the coronal plane, a stereomicroscope was used for general observation of the regenerated tissue, and SEM (Phenom-World Co., Ltd., Netherlands) was used to observe the microstructure.

### Histological staining and immunostaining

Rabbit jawbones were decalcified with 10% acetic acid buffer (pH 8.0) for 4 months, embedded in paraffin and then sectioned at 5 µm. The tissue sections were routinely deparaffinized and hydrated, incubated in sodium citrate solution for tissue antigen retrieval, and then incubated with the blocking solution at room temperature for 20 min to block endogenous peroxidase activity. The samples were subsequently blocked with normal goat serum for 40 min at room temperature, followed by incubation with primary antibodies overnight at 4 °C. Finally, a 3′-diaminobenzidine (DAB) kit was used to detect antigen expression in the DPSCs and DPSCs/PDL groups. The primary antibodies used were as follows: rabbit anti-dentin sialophosphoprotein (DSPP; bs-10316R; Bioss) and rabbit anti-dentin matrix acidic phosphoprotein 1 (DMP1; bs-12359R; Bioss). Conventional haematoxylin and eosin (H&E) staining and microscopy (OLYMPUS BX53) were used for histomorphological analysis in the blank control, DPSCs and DPSCs/PDL groups.

### RNA-seq and bioinformatic analysis in vitro

We next aimed to explore the mechanism by which residual periodontal ligament promotes the dentin regeneration potential of DPSCs in the jaw. We cocultured DPSCs and PDLSCs in vitro and compared the gene expression profiles of DPSCs cocultured with and cultured without PDLSCs to identify differentially expressed genes. DPSCs and PDLSCs were cocultured in six-well Transwell plates, separated by a 0.4-µm pore-size filter membrane. DPSCs were collected after a total of 3 days of coculture, and TRIzol reagent (Invitrogen) was used to extract total RNA. The RNA quantity and quality were determined by a multiImager and spectrophotometer (Meriton, China). Then, according to the manufacturer’s instructions, a library was constructed using TruSeq Stranded Total RNA with Ribo-Zero Gold (Illumina). Transcriptome sequencing and analysis were performed by OE Biotech Co., Ltd. DESeq was used to screen for differentially expressed genes according to the conditions of *q* < 0.05 and fold change > 2 or fold change < 0.5. After identifying the differentially expressed genes, Gene Ontology (GO) enrichment analysis was performed to describe their functions, and the Kyoto Encyclopedia of Genes and Genomes (KEGG) database was used to perform a pathway analysis.

### Statistical analysis

All statistical analyses were performed with SPSS 22 statistical software. The statistical significance of the differences was determined by Student’s t-test, including for comparison of the volume of dentin-like tissue and expression of DMP1 and DSPP in the DPSCs and DPSCs/PDL groups and the expression of mRNAs in the DPSCs and DPSCs/PDLSCs groups. *P* < 0.05 was considered significant.

## Results

### Expression of surface markers on rabbit DPSCs

**Fig. 1:**
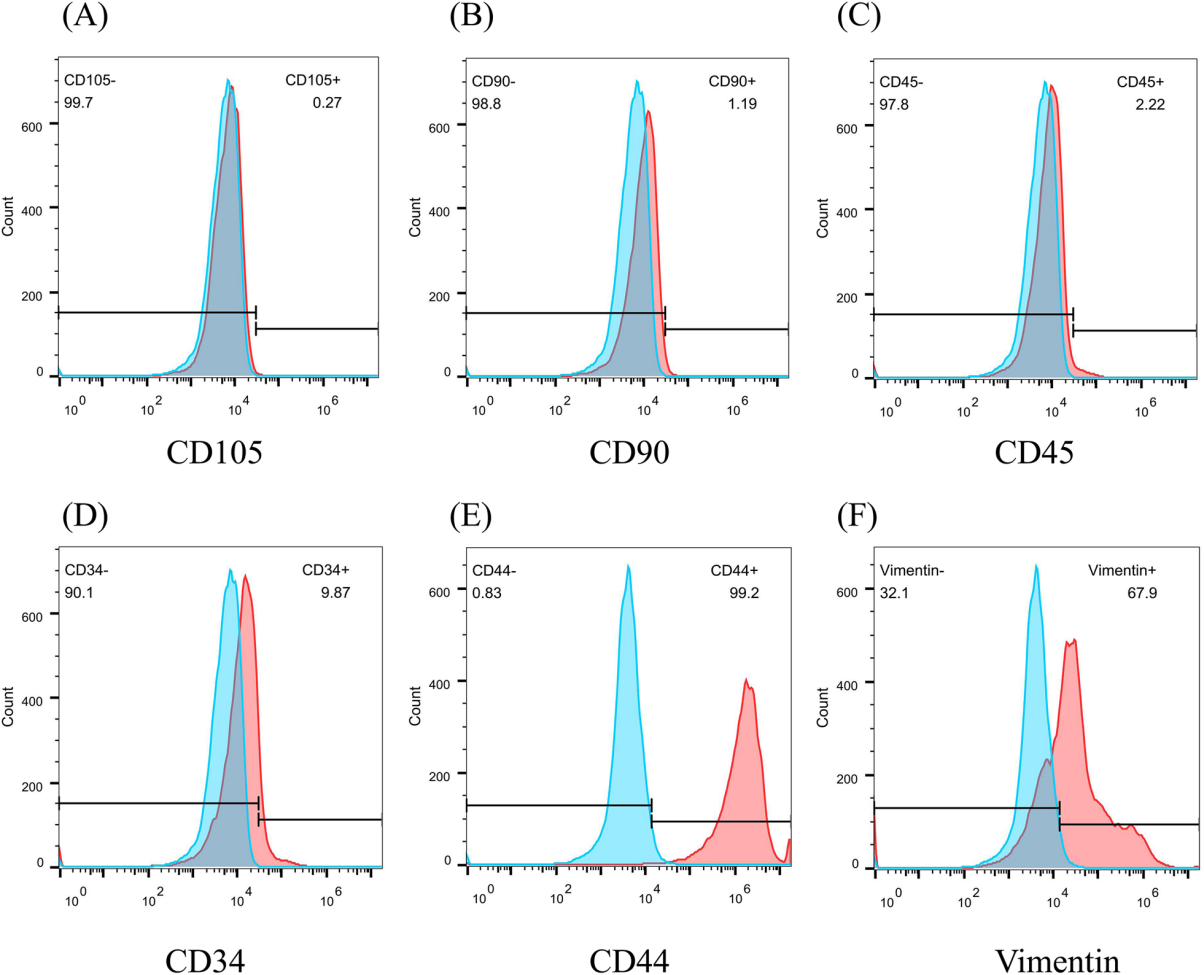
Rabbit DPSCs surface marker expression. Rabbit DPSCs negatively expressed **A** CD105, **B** CD90, **C** CD45 and **D** CD34 and positively expressed **E** CD44 and **F** vimentin

Flow cytometry was used to identify the surface markers of rabbit DPSCs. The results showed positive expression of CD44 and vimentin and negative expression of CD34, CD45, CD90, and CD105, indicating rabbit DPSCs (Fig. 1).

### Residual periodontal ligament promotes the dentin regeneration potential of DPSCs in the rabbit jaw

**Fig. 2:**
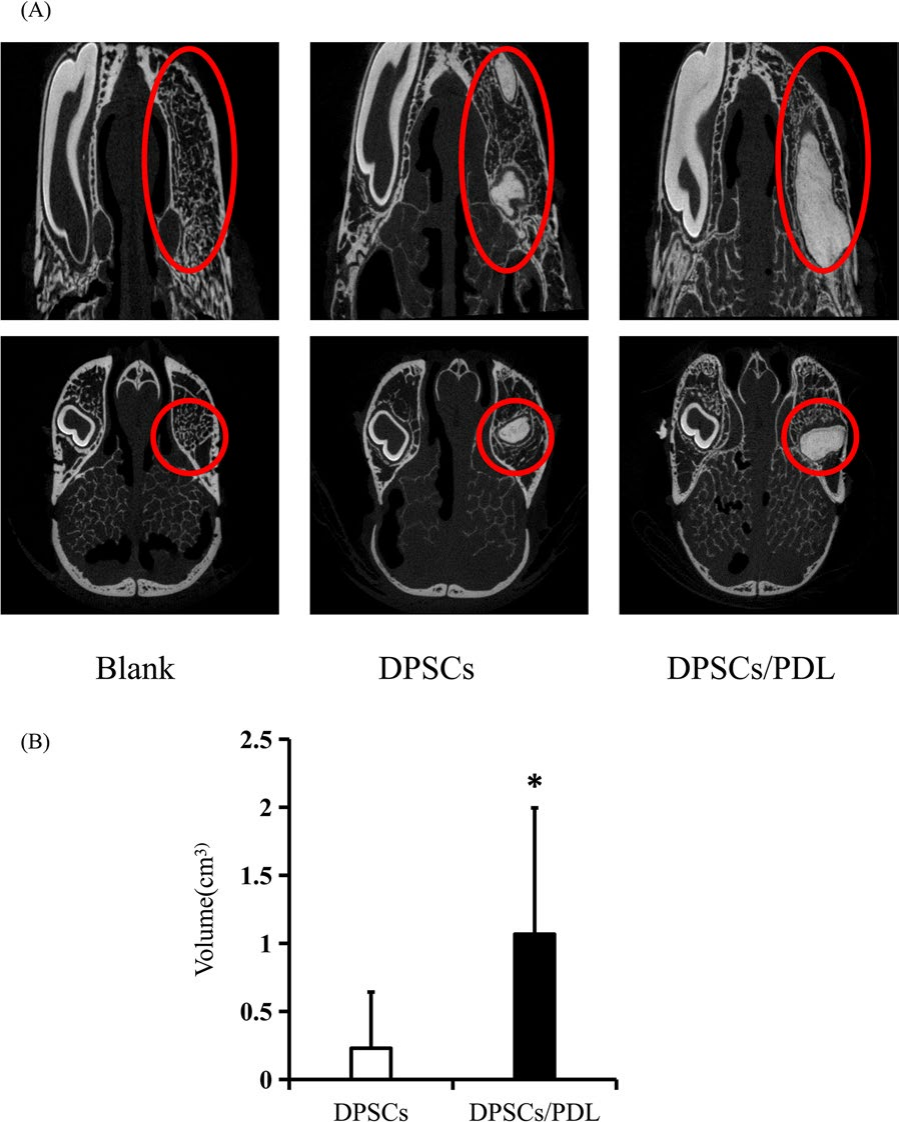
Micro-CT findings. **A** Micro-CT showed complete bone-like healing in the blank control group, a small number of sites of high-density dentin-like tissue in the DPSCs group, and larger-volume sites of high-density dentin-like tissue in the DPSCs/PDL group. **B** The volume of the regions of regenerated high-density tissue was larger in the DPSCs/PDL group than in the DPSCs group. Statistical significance was determined by Student’s t-test. SD is represented by bars. *P < 0.05

Rabbit jawbones were obtained 3 months after the model was established and examined by micro-CT. The results showed complete bone-like healing without any high-density shadows in the blank control group. In the DPSCs group, high-density dentin-like tissue was observed at a small number of sites (3/8), while in the DPSCs/PDL group, more sites (6/8) and larger volumes of high-density dentin-like tissue were observed. Subsequently, we used CTan software to perform three-dimensional reconstruction of the micro-CT images and volume calculation of the high-density regions of regeneration. The volume of the high-density regions of regeneration in the DPSCs/PDL group (1.07 ± 0.93 cm^3^) was larger than that in the DPSCs group (0.23 ± 0.41 cm^3^), and the difference was statistically significant (*P* = 0.04) (Fig. 2).

**Fig. 3:**
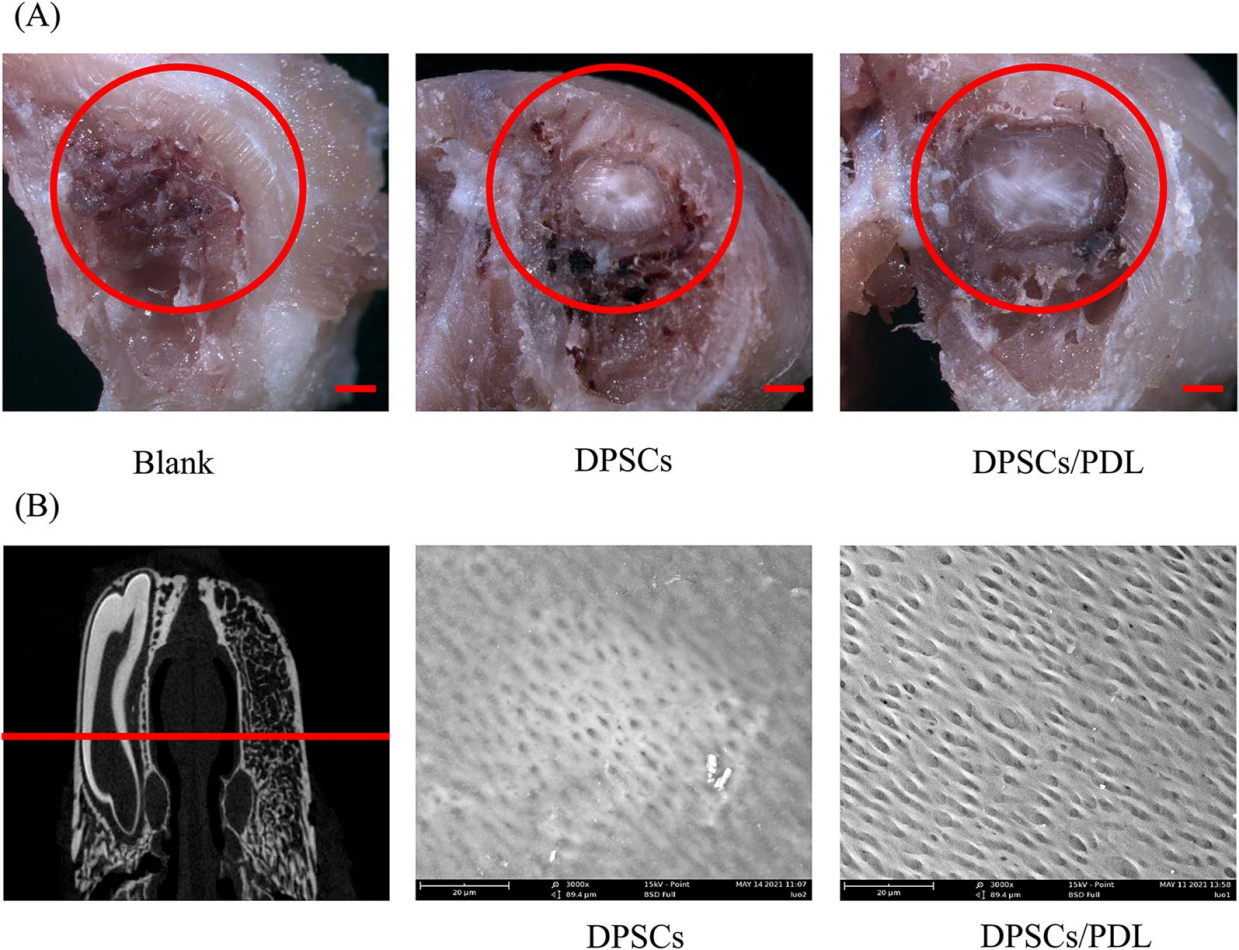
Stereomicroscopy and SEM findings. **A** The general morphology of the tissue was observed by stereomicroscopy. Complete bone healing without any dentin-like tissue was observed in the blank control group; small areas of dentin-like tissue were observed in the DPSCs group; and larger areas of dentin-like tissue were observed in the DPSCs/PDL group. **B** SEM revealed regenerated dentin-like tissue in the DPSCs group and dentin tubules in addition to regenerated dentin-like tissue in the DPSCs/PDL group

The results of stereomicroscope were similar to those of micro-CT. The blank control group showed complete bone healing without any dentin-like tissue; small areas of dentin-like tissue were observed in the DPSCs group; and larger areas of dentin-like tissue were observed in the DPSCs/PDL group (Fig. 3A). Finally, the microstructure of the regenerated dentin-like tissue was observed by SEM. These results showed that the regenerated dentin-like tissue in the DPSCs and DPSCs/PDL groups contained dentin tubules, confirming that the regenerated tissue was dentin. We also noticed that compared with the regenerated dentin tubules in the DPSCs group, those in the DPSCs/PDL group were more regular and had clearer structures (Fig. 3B).

**Fig. 4:**
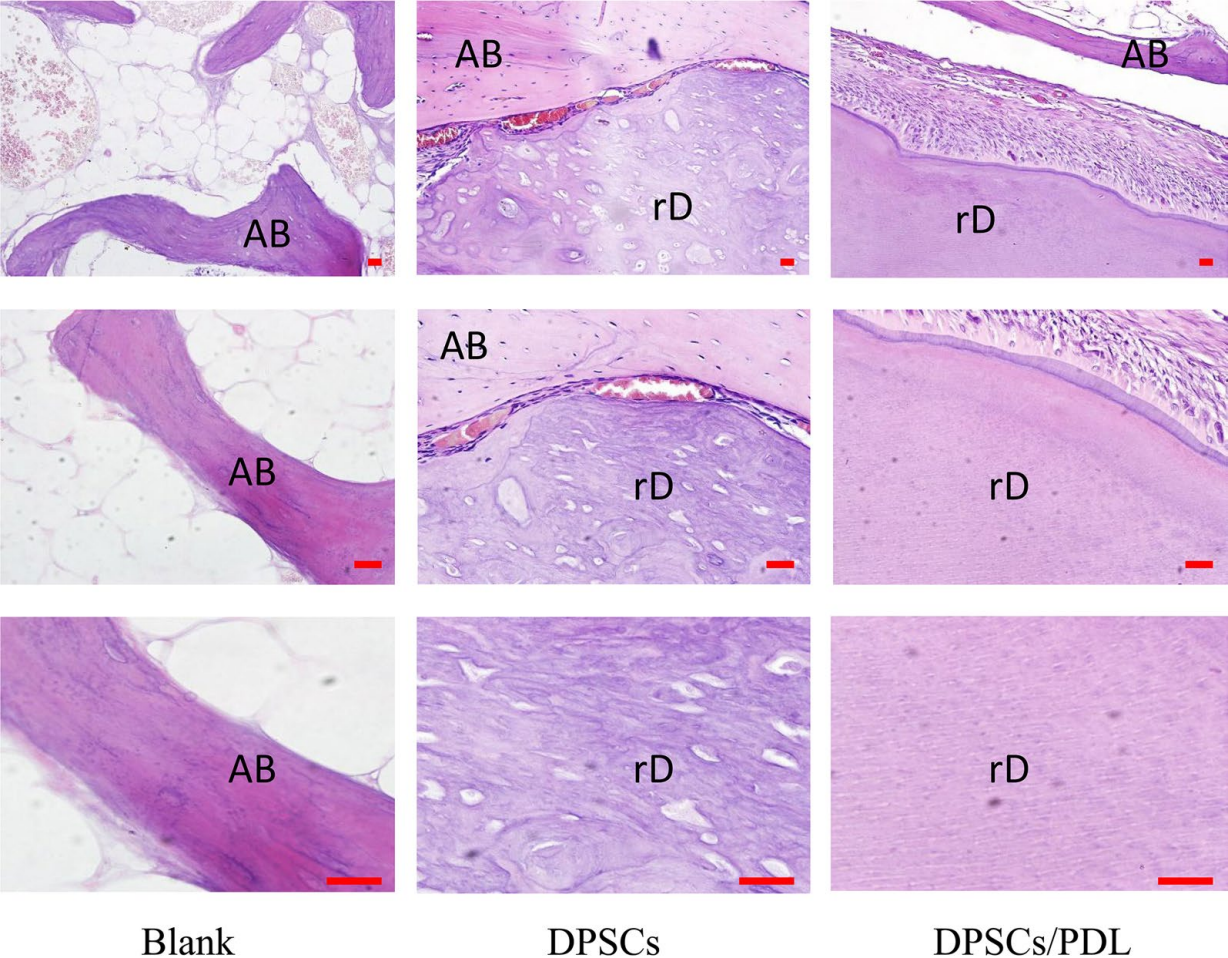
H&E staining results. The regenerated tissue in the blank control group was bone tissue. The regenerated tissue in the DPSCs group as similar to dentin, with irregular dentin tubules visible. The dentin tubules in the DPSCs/PDL group were arranged regularly and surrounded by odontoblast-like cells

**Fig. 5:**
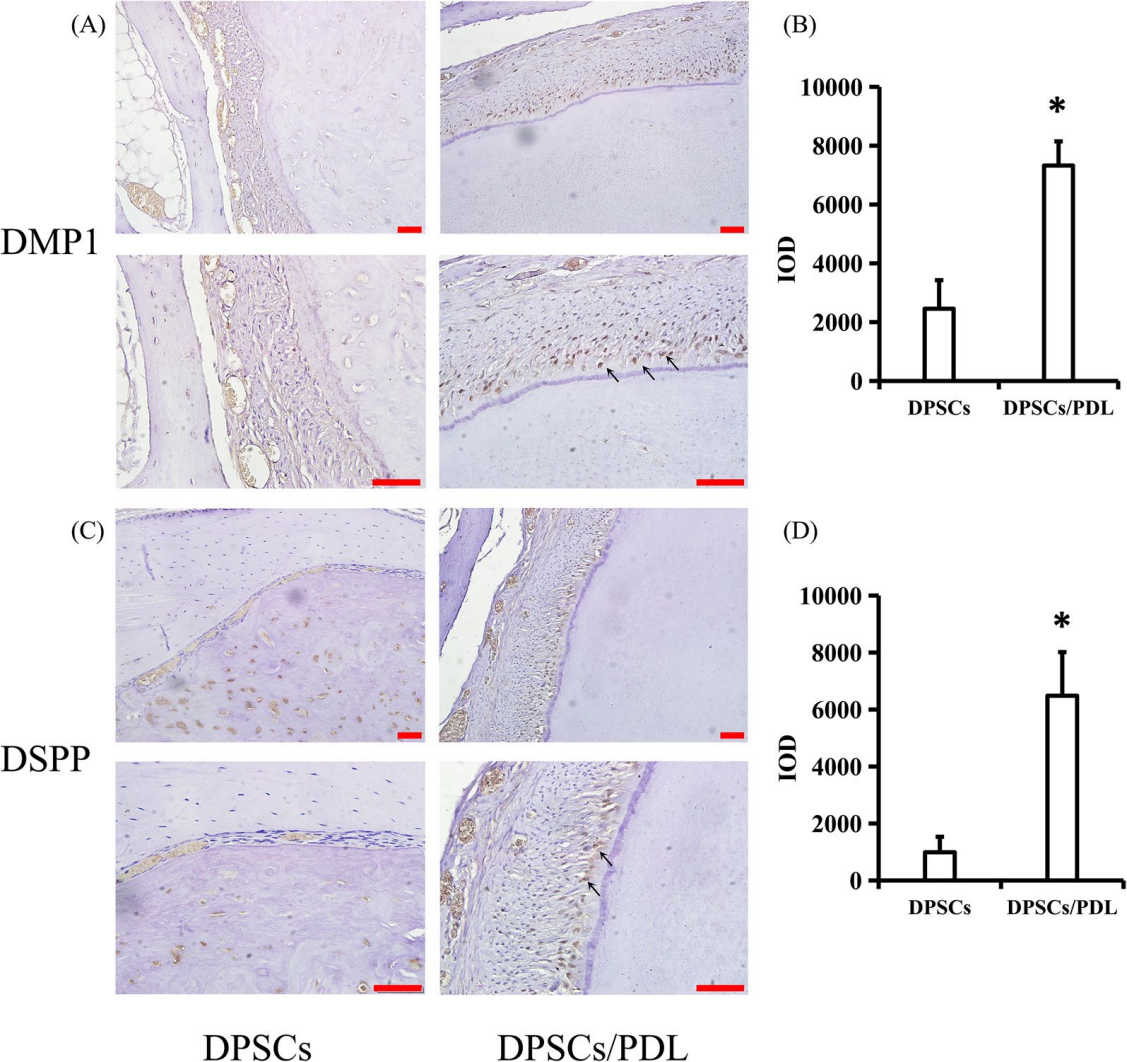
Immunohistochemical staining results. **A**, **B** DMP1- and **C**, **D** DSPP-positive cells were significantly more abundant in the DPSCs/PDL group than in the DPSCs group. Black arrow: DMP1- or DSPP-positive cells. Statistical significance was determined by Student’s t-test. SD is represented by bars. *P < 0.05

H&E staining showed that the regenerated tissue in the blank control group was bone tissue, while the dentin-like tissue observed in the DPSCs and DPSCs/PDL groups were surrounded by odontoblasts. In the DPSCs group, the regenerated dentin structure was chaotic, the dentin tubules were irregular, and there were fewer surrounding odontoblasts. However, the regenerated dentin structure in the DPSCs/PDL group was regular, and the dentin tubules were clear in shape, arranged neatly, and surrounded by a large number of odontoblasts (Fig. 4). To confirm that the regenerated high-density tissue was dentin, we used immunohistochemical staining to detect dentin-specific proteins in the regenerated tissue. The results showed significantly more DMP1- and DSPP-positive cells in the DPSCs/PDL group than in the DPSCs group (DMP1: *P* = 0.02, DSPP: *P* = 0.01) (Fig. 5).

### Identification of differentially expressed genes in DPSCs after coculture with PDLSCs in vitro

**Fig. 6:**
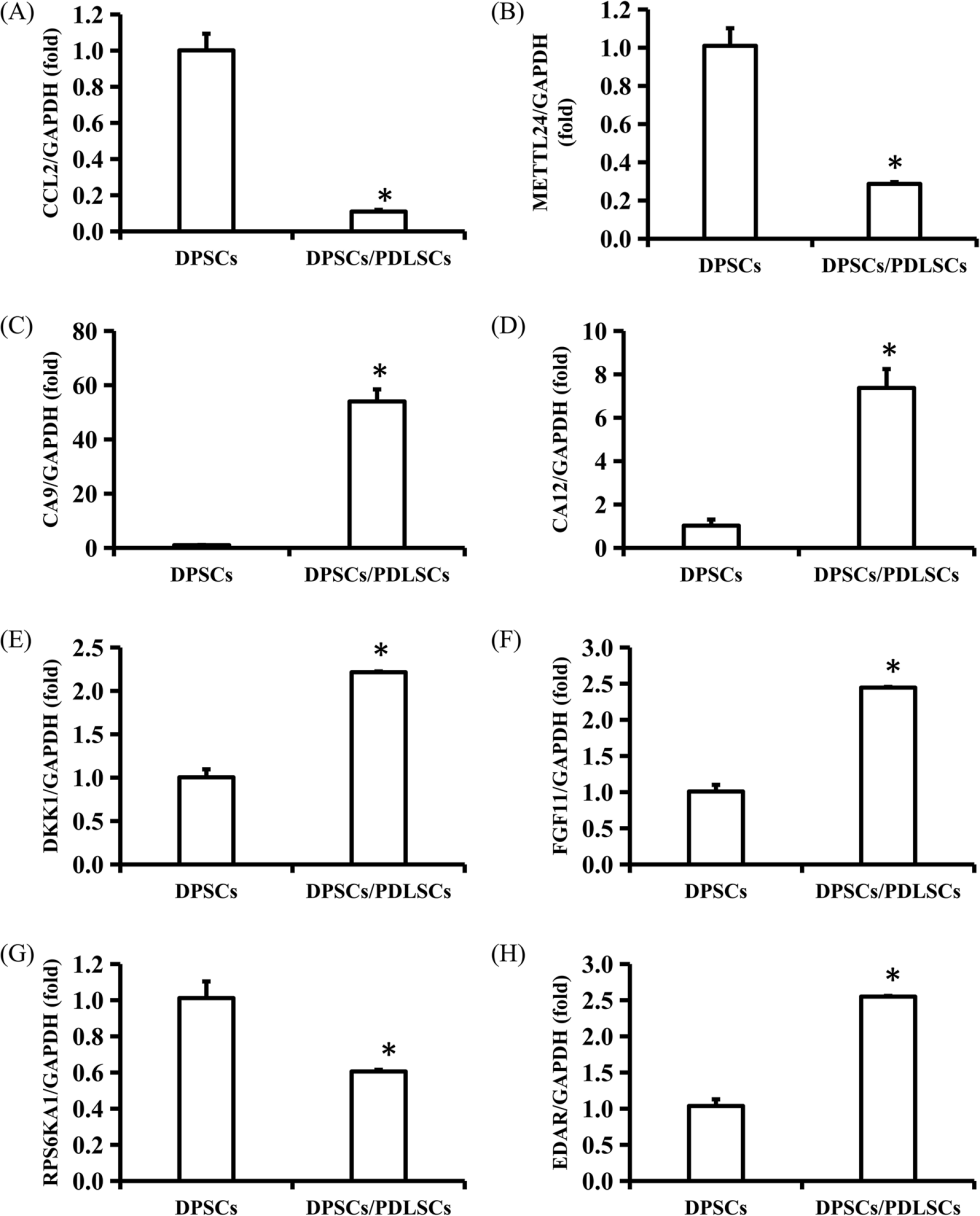
Cocultivation with PDLSCs altered DPSCs gene expression levels. **A**–**D** Cocultivation of PDLSCs and DPSCs resulted in downregulation of CCL2 and METTL24 and upregulation of CA9 and CA12 in DPSCs. **E**–**H** Cocultivation of PDLSCs and DPSCs resulted in upregulation of DKK1, FGF11 and EDAR and downregulation of RPS6KA1 in DPSCs. GAPDH was used as an internal reference. Statistical significance was determined by Student’s *t* test. SD is represented by bars. *P < 0.05

Using *P* < 0.05 and fold change > 2.0 or fold change < 0.5 as screening criteria, 427 differentially expressed mRNAs were detected; 285 mRNAs were upregulated, and 142 mRNAs were downregulated (see Additional file 2). Forty-one differentially expressed lncRNAs were detected; 26 lncRNAs were upregulated, and 15 lncRNAs were downregulated (see Additional file 3). A total of 411 differentially expressed circRNAs were detected; 224 circRNAs were upregulated, and 187 circRNAs were downregulated (see Additional file 4). Nineteen differentially expressed miRNAs were detected, including 13 upregulated miRNAs and 6 downregulated miRNAs (see Additional file 5). Among the differentially expressed genes, we selected the 4 genes (CCL2, METTL24, CA9, CA12) with the largest fold changes and 4 genes (DKK1, FGF11, RPS6KA1, EDAR) related to dentinogenic differentiation pathways to verify the accuracy and credibility of the microarray results. The real-time RT–PCR results showed that the cocultivation of PDLSCs and DPSCs resulted in the downregulation of CCL2, METTL24 and RPS6KA1 and the upregulation of CA9, CA12, DKK1, FGF11 and EDAR in DPSCs, consistent with the RNA-seq results (Fig. 6 and Additional file 1).

**Fig. 7:**
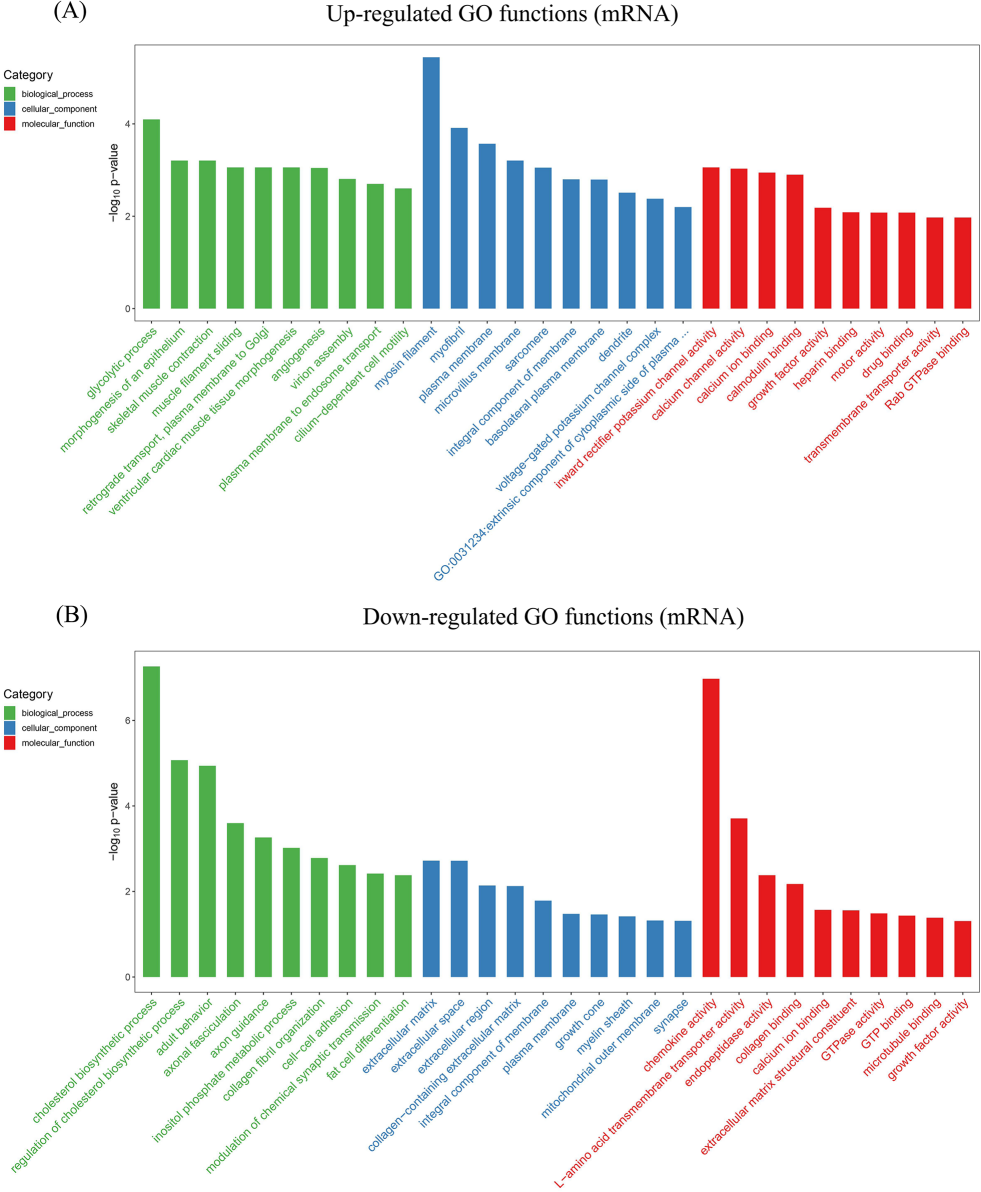
GO functional enrichment analysis of differentially expressed mRNAs. **A** Top 30 upregulated GO functions of the differentially expressed mRNAs. **B** Top 30 downregulated GO functions of the differentially expressed mRNAs

The GO functional enrichment analysis of differentially expressed mRNAs, lncRNAs, circRNAs and miRNA target mRNAs was used to investigate function in terms of three aspects, i.e., biological process, cellular component and molecular function. The GO enrichment analysis of differentially expressed mRNAs revealed 198 upregulated GO functions and 80 downregulated GO functions (see Additional file 6). The upregulated GO functions included glycolytic process, myosin filament and inward rectifier potassium channel activity (Fig. 7A). The downregulated GO functions included cholesterol biosynthetic process, extracellular matrix and chemokine activity (Fig. 7B). The GO enrichment analysis of differentially expressed lncRNAs revealed 109 upregulated GO functions and 130 downregulated GO functions (see Additional file 7). The upregulated GO functions included biological regulation, cell and binding (see Additional file 8). The downregulated GO functions included biological adhesion, cell and binding (see Additional file 9). The GO enrichment analysis of differentially expressed circRNAs revealed 111 upregulated GO functions and 161 downregulated GO functions (see Additional file 10). The upregulated GO functions included biological adhesion, cell and binding (see Additional file 11). The downregulated GO functions included biological adhesion, cell and binding (see Additional file 12). The GO enrichment analysis of differentially expressed miRNA target mRNAs revealed 3124 differentially regulated GO functions (see Additional file 13). The differentially regulated GO functions included biological adhesion, cell and antioxidant activity (see Additional file 14).

**Fig. 8:**
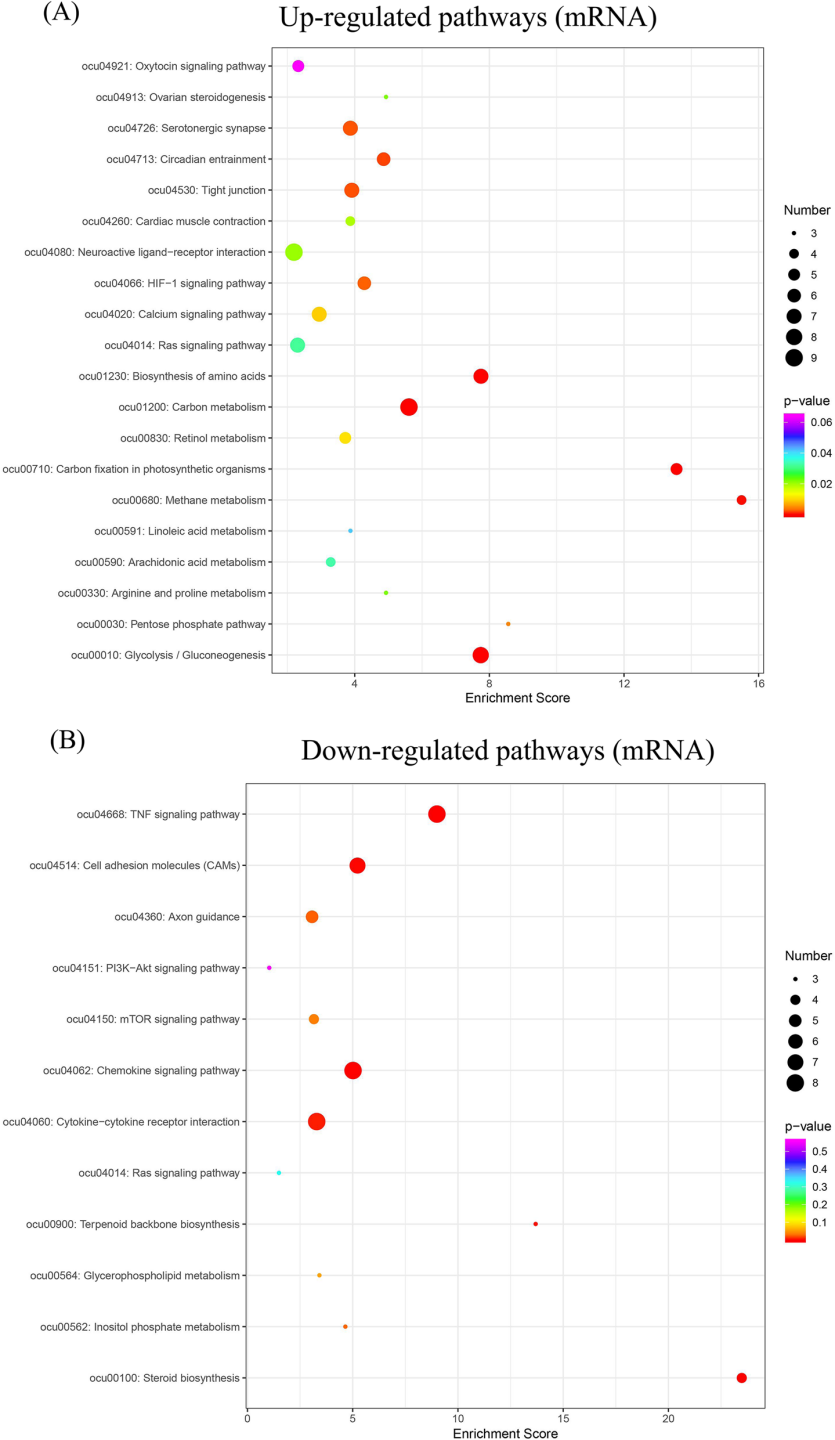
Pathway analysis of differentially expressed mRNAs using the KEGG database. **A** Top 20 upregulated pathways of the differentially expressed mRNAs. **B** Top 20 downregulated pathways of the differentially expressed mRNAs

Pathway analysis of differentially expressed mRNAs, lncRNAs, circRNAs and miRNA target mRNAs was performed using the KEGG database. Differentially expressed mRNAs revealed a total of 118 upregulated pathways and 119 downregulated pathways (see Additional file 15). The upregulated pathways included glycolysis/gluconeogenesis, carbon fixation in photosynthetic organisms and carbon metabolism (Fig. 8A). The downregulated pathways included the tumour necrosis factor (TNF) signalling pathway, steroid biosynthesis and chemokine signalling pathways (Fig. 8B). Differentially expressed lncRNAs revealed a total of 46 upregulated pathways and 37 downregulated pathways (see Additional file 16). The upregulated pathways included galactose metabolism, starch and sucrose metabolism and inositol phosphate metabolism (see Additional file 17). The downregulated pathways included the AMPK signalling pathway, the TGF-β signalling pathway and fc gamma R-mediated phagocytosis (see Additional file 18). Differentially expressed circRNAs revealed a total of 102 upregulated pathways and 119 downregulated pathways (see Additional file 19). The upregulated pathways included the apoptosis—fly, HIF-1 signalling and p53 signalling pathways (see Additional file 20). The downregulated pathways included the MAPK signalling pathway—fly, alanine, aspartate and glutamate metabolism and cholinergic synapse (see Additional file 21). Differentially expressed miRNA target mRNAs revealed a total of 238 differentially regulated pathways (see Additional file 22). The differentially regulated pathways included axon guidance, the oestrogen signalling pathway and actin cytoskeleton regulation (see Additional file 23).

## Discussion

With the development of stem cell tissue engineering technology, significant breakthroughs in tooth regeneration mediated by odontogenic stem cells have been achieved in recent years, but there are still many problems that need to be resolved in the field of tooth regeneration [28, 29]. In this study, we established a tooth regeneration model in the rabbit jaw in which residual periodontal ligament is retained in the fresh extraction socket. The results show that the residual periodontal ligament microenvironment in the extraction socket can promote the dentin regeneration potential of DPSCs in the rabbit jaw.

The cellular microenvironment supports and maintains the proliferation, differentiation and regeneration potential of MSCs. A large number of studies have clarified the importance of the MSC microenvironment [30, 31]. The cellular microenvironment is also an important factor that determines cell behaviour and tooth morphogenesis. In tooth regeneration research, stem cells, ECM, growth factors and multiple interactions among them determine the formation, development and eruption of teeth [32, 33]. Our results show that the residual periodontal ligament microenvironment in the rabbit jaw is conducive to dentin regeneration by DPSCs in terms of improving the efficiency of DPSCs-mediated dentin regeneration, producing regenerated dentin with a more regular structure, yielding dentin tubules with a more orderly arrangement, and increasing the expression of the dentin-specific proteins DMP1 and DSPP.

Periodontal ligament is a natural connective tissue existing between the tooth root and alveolar bone and is mainly composed of periodontal fibrous ligament, cells and ECM [24, 25]. After a tooth is extracted from the jaw, some periodontal ligament tissue will remain on the inner wall of the extraction socket [34]. After DPSCs are implanted in the extraction socket, the periodontal ligament tissue becomes the odontogenic cellular microenvironment surrounding the implanted MSCs, which regulates the regeneration potential and differentiation of DPSCs. The ECM is a noncellular component of tissue and a highly organized and complex structure composed of structural and functional proteins [35]. Each tissue has unique ECM characteristics, which provide guiding cues for cell differentiation, cell migration, wound healing and immune responses. In short, the ECM determines cell and tissue function. A recent study found that the ECM contains a large number of growth factors and proteins related to MSC differentiation, including VEGF, RUNX2, and BMP2 [23]. In the RNA-seq results of this study, we found 427 differentially expressed protein-coding RNAs; 85 mRNAs were upregulated, and 142 mRNAs were downregulated (see Additional file 2). According to the bioinformatic analysis, we found some of the differentially expressed genes, including MMP13, TIMP4, FBN2, WISP1, TNXB, and ADAMTS3, are related to the ECM. These ECM regulators are widely involved in the composition and conversion of ECM [37, 38]. MMPs are a class of metal ion-dependent proteolytic enzymes, and TIMPs are their inhibitors. The main function of MMPs is to degrade the ECM, and they can also activate growth factors and adhesion molecule enzymes. The MMP/TIMP system is the most important enzyme system regulating the dynamic balance of the ECM, and it plays an important role in the development of inflammation in various tissues, the formation of new blood vessels and the regeneration of tissue. Studies have found that MMPs/TIMPs participate in tooth movement, tissue regeneration and tissue remodelling by maintaining ECM homeostasis [36–38]. Fibrillins (FBNs) are structural components of the ECM that serve to distribute, concentrate and regulate local TGF-β and BMP signals, which regulate numerous cellular activities, including ECM formation and remodelling and tissue regeneration [39, 40]. TGF-β and BMP have been proven to promote the dentinogenic differentiation of DPSCs [41]. A study combining TGF-β and BMP into bioscaffold materials demonstrated that the combination promoted the regeneration of biological tooth roots [42]. WISP1 is a connective tissue growth factor and a target of the wnt/frizzled pathway [43], and the wnt pathway is an important regulatory pathway for dentinogenic MSC differentiation. In addition, the identified ECM-related differentially expressed genes include a variety of genes for collagen, TNXB and ADAMTS-1, which may also be involved in the beneficial effect of residual periodontal ligament on DPSCs differentiation. In conclusion, residual periodontal ligament in the extraction socket may be used as a kind of odontogenic ECM to regulate the dentinogenic differentiation of DPSCs in the jaw through a variety of factors.

To explore the specific mechanism by which residual periodontal ligament promotes the dentin regeneration potential of DPSCs in the jaw, we cocultured PDLSCs isolated from the periodontal ligament with DPSCs. Then, transcriptome sequencing of the DPSCs was performed to determine the differentially expressed gene profile, and GO enrichment and KEGG pathway analyses of the differentially expressed genes were carried out. The GO functions related to the influence of PDLSCs on DPSCs included glycolytic process, ECM and chemokine activity. These findings are consistent with those of our previous analysis, indicating that the ECM in residual periodontal ligament may be used as a microenvironment to regulate the process of DPSCs differentiation.

The differentially regulated pathways are related to the influence of PDLSCs on DPSCs. The upregulated pathways included glycolysis/gluconeogenesis, carbon fixation in photosynthetic organisms and carbon metabolism, while the downregulated pathways included the TNF signalling pathway, steroid biosynthesis and chemokine signalling pathways. TNF-α is a cytokine with pleiotropic biological effects that can affect the differentiation of MSCs [44–46]. In this study, it was found that PDLSCs negatively regulate TNF signalling in DPSCs, potentially promoting the dentinogenic differentiation of DPSCs. Chemokines are small cytokines or signal proteins secreted by cells. MSCs from different sources have the ability to secrete different chemokines and are regulated by chemokines [47]. The differentiation of MSCs is the result of the interaction of multiple signalling pathways. These differentially regulated signalling pathways jointly regulate the dentinogenic differentiation of DPSCs in the jaw. However, much research is still needed to further explore the possible mechanisms.

The microenvironment is involved in determining the differentiation fate of cells. In this study, an odontogenic microenvironment was created by retaining residual periodontal ligament tissue in the extraction socket, and this odontogenic microenvironment was applied in an animal model of tooth regeneration. The results show that the periodontal ligament microenvironment promotes the dentin regeneration potential of DPSCs in the jaw, which provides a theoretical basis for the study of tooth regeneration. However, because the residual periodontal ligament in the extraction socket could not be completely separated for in vitro studies, the in vivo situation cannot be completely simulated in vitro. In the future, other methods are needed to simulate the periodontal ligament microenvironment in vitro to explore not only potential mechanisms in more detail but also ways to enhance their effects.

## Conclusions

In conclusion, our results indicate that residual periodontal ligament can promote the dentin regeneration potential of DPSCs in the jaw. RNA-seq and bioinformatic analysis revealed that ECM, TNF signalling and chemokine signalling pathways may represent key factors and signalling pathways through which residual periodontal ligament promotes the dentin regeneration potential of DPSCs. These discoveries provide new insights for further research on MSC-mediated tooth regeneration.

## Supplementary Information

**Additional file 1**: Primers for specific genes.

**Additional file 2**: Differentially expressed mRNAs in DPSCs regulated by PDLSCs.

**Additional file 3**: Differentially expressed lncRNAs in DPSCs regulated by PDLSCs.

**Additional file 4**: Differentially expressed circRNAs in DPSCs regulated by PDLSCs.

**Additional file 5**: Differentially expressed miRNAs in DPSCs regulated by PDLSCs.

**Additional file 6**: GO functional enrichment analysis of differentially expressed mRNAs in DPSCs regulated by PDLSCs.

**Additional file 7**: GO functional enrichment analysis of differentially expressed lncRNAs in DPSCs regulated by PDLSCs.

**Additional file 8**: Upregulated GO functions of differentially expressed lncRNAs in DPSCs regulated by PDLSCs.

**Additional file 9**: Downregulated GO functions of differentially expressed lncRNAs in DPSCs regulated by PDLSCs.

**Additional file 10**: GO functional enrichment analysis of differentially expressed circRNAs in DPSCs regulated by PDLSCs.

**Additional file 11**: Upregulated GO functions of differentially expressed circRNAs in DPSCs regulated by PDLSCs.

**Additional file 12**: Downregulated GO functions of differentially expressed circRNAs in DPSCs regulated by PDLSCs.

**Additional file 13**: GO functional enrichment analysis of differentially expressed miRNA target mRNAs in DPSCs regulated by PDLSCs.

**Additional file 14**: GO functions of differentially expressed miRNA target mRNAs in DPSCs regulated by PDLSCs.

**Additional file 15**: KEGG enrichment analysis of differentially expressed mRNAs in DPSCs regulated by PDLSCs.

**Additional file 16**: KEGG enrichment analysis of differentially expressed lncRNAs in DPSCs regulated by PDLSCs.

**Additional file 17**: Upregulated pathways of differentially expressed lncRNAs in DPSCs regulated by PDLSCs.

**Additional file 18**: Downregulated pathways of differentially expressed lncRNAs in DPSCs regulated by PDLSCs.

**Additional file 19**: KEGG enrichment analysis of differentially expressed circRNAs in DPSCs regulated by PDLSCs.

**Additional file 20**: Upregulated pathways of differentially expressed circRNAs in DPSCs regulated by PDLSCs.

**Additional file 21**: Downregulated pathways of differentially expressed circRNAs in DPSCs regulated by PDLSCs.

**Additional file 22**: KEGG enrichment analysis of differentially expressed miRNA target mRNAs in DPSCs regulated by PDLSCs.

**Additional file 23**: Pathways of differentially expressed miRNA target mRNAs in DPSCs regulated by PDLSCs.

## Data Availability

RNA-seq datasets have been uploaded to the Genome Sequence Archive (GSA No: CRA006856, Project: PRJCA009431). Other data generated or analysed during this study are included in this published article [and its additional files].

## Abbreviations

MSCs: Mesenchymal stem cells
DPSCs: Dental pulp stem cells
BMP2: Bone morphogenetic protein 2
SFRP2: Secreted frizzled-related protein 2
ECM: Extracellular matrix
PDL: Periodontal ligament
PBS: Phosphate-buffered saline
SEM: Scanning electron microscopy
DSPP: Dentin sialophosphoprotein
DMP1: Dentin matrix acidic phosphoprotein 1
PDLSCs: Periodontal ligament stem cells
TNF: Tumour necrosis factor

## References

[1] Cooper LF (2009). The current and future treatment of edentulism. J Prosthodont.

[2] Mark AM (2020). Preventing tooth loss. J Am Dent Assoc.

[3] Donovan TE (2006). Longevity of the tooth/restoration complex: a review. J Calif Dent Assoc.

[4] Mozaffari MS, Emami G, Khodadadi H, Baban B (2019). Stem cells and tooth regeneration: prospects for personalized dentistry. EPMA J.

[5] Lee DJ, Lee JM, Kim EJ, Takata T, Abiko Y, Okano T (2017). Bio-implant as a novel restoration for tooth loss. Sci Rep.

[6] Li H, Sun J, Yang H, Han X, Luo X, Liao L (2021). Recruited CD68(+)CD206(+) macrophages orchestrate graft immune tolerance to prompt xenogeneic-dentin matrix-based tooth root regeneration. Bioact Mater.

[7] Nakahara T (2011). Potential feasibility of dental stem cells for regenerative therapies: stem cell transplantation and whole-tooth engineering. Odontology.

[8] Sonoyama W, Liu Y, Fang D, Yamaza T, Seo BM, Zhang C (2006). Mesenchymal stem cell-mediated functional tooth regeneration in swine. PLoS ONE.

[9] Wei F, Song T, Ding G, Xu J, Liu Y, Liu D (2013). Functional tooth restoration by allogeneic mesenchymal stem cell-based bio-root regeneration in swine. Stem Cells Dev.

[10] Gao ZH, Hu L, Liu GL, Wei FL, Liu Y, Liu ZH (2016). Bio-root and implant-based restoration as a tooth replacement alternative. J Dent Res.

[11] Hu L, Liu Y, Wang S (2018). Stem cell-based tooth and periodontal regeneration. Oral Dis.

[12] Wang C, Wang Y, Wang H, Yang H, Cao Y, Xia D (2021). SFRP2 enhances dental pulp stem cell-mediated dentin regeneration in rabbit jaw. Oral Dis.

[13] Hung CN, Mar K, Chang HC, Chiang YL, Hu HY, Lai CC (2011). A comparison between adipose tissue and dental pulp as sources of MSCs for tooth regeneration. Biomaterials.

[14] Lane SW, Williams DA, Watt FM (2014). Modulating the stem cell niche for tissue regeneration. Nat Biotechnol.

[15] Wagers AJ (2012). The stem cell niche in regenerative medicine. Cell Stem Cell.

[16] Crisan M, Yap S, Casteilla L, Chen CW, Corselli M, Park TS (2008). A perivascular origin for mesenchymal stem cells in multiple human organs. Cell Stem Cell.

[17] Voog J, Jones DL (2010). Stem cells and the niche: a dynamic duo. Cell Stem Cell.

[18] Sui BD, Hu CH, Liu AQ, Zheng CX, Xuan K, Jin Y (2019). Stem cell-based bone regeneration in diseased microenvironments: challenges and solutions. Biomaterials.

[19] Sui BD, Hu CH, Zheng CX, Jin Y (2016). Microenvironmental views on mesenchymal stem cell differentiation in aging. J Dent Res.

[20] Sui B, Hu C, Liao L, Chen Y, Zhang X, Fu X (2016). Mesenchymal progenitors in osteopenias of diverse pathologies: differential characteristics in the common shift from osteoblastogenesis to adipogenesis. Sci Rep.

[21] Sui BD, Hu CH, Zheng CX, Shuai Y, He XN, Gao PP (2017). Recipient glycemic micro-environments govern therapeutic effects of mesenchymal stem cell infusion on osteopenia. Theranostics.

[22] Ko KI, Coimbra LS, Tian C, Alblowi J, Kayal RA, Einhorn TA (2015). Diabetes reduces mesenchymal stem cells in fracture healing through a TNFalpha-mediated mechanism. Diabetologia.

[23] Gao CY, Huang ZH, Jing W, Wei PF, Jin L, Zhang XH (2018). Directing osteogenic differentiation of BMSCs by cell-secreted decellularized extracellular matrixes from different cell types. J Mater Chem B.

[24] Berkovitz BK (2004). Periodontal ligament: structural and clinical correlates. Dent Update.

[25] Shuttleworth CA, Smalley JW (1983). Periodontal ligament. Int Rev Connect Tissue Res.

[26] Hao J, Yang H, Cao Y, Zhang C, Fan Z (2020). IGFBP5 enhances the dentinogenesis potential of dental pulp stem cells via JNK and ErK signalling pathways. J Oral Rehabil.

[27] Qin Q, Yang H, Zhang C, Han X, Guo J, Fan Z (2021). lncRNA HHIP-AS1 promotes the osteogenic differentiation potential and inhibits the migration ability of periodontal ligament stem cells. Stem Cells Int.

[28] Cheng N, Wen J, Hitching R, Lei C, Xu C (2021). Tooth bioengineering and whole tooth regeneration: regenerative approaches in dentistry.

[29] Baranova J, Buchner D, Gotz W, Schulze M, Tobiasch E (2020). Tooth formation: are the hardest tissues of human body hard to regenerate?. Int J Mol Sci.

[30] Donnelly H, Salmeron-Sanchez M, Dalby MJ (2018). Designing stem cell niches for differentiation and self-renewal. J R Soc Interface.

[31] Bloom AB, Zaman MH (2014). Influence of the microenvironment on cell fate determination and migration. Physiol Genom.

[32] Zheng C, Chen J, Liu S, Jin Y (2019). Stem cell-based bone and dental regeneration: a view of microenvironmental modulation. Int J Oral Sci.

[33] Zhai Q, Dong Z, Wang W, Li B, Jin Y (2019). Dental stem cell and dental tissue regeneration. Front Med.

[34] Wang L, Shen H, Zheng W, Tang L, Yang Z, Gao Y (2011). Characterization of stem cells from alveolar periodontal ligament. Tissue Eng Part A.

[35] Bronckers ALJJ, Lyaruu DM, Wöltgens JHM (1989). Immunohistochemistry of extracellular matrix proteins during various stages of dentinogenesis. Connect Tissue Res.

[36] Leonardi R, Talic NF, Loreto C (2007). MMP-13 (collagenase 3) immunolocalisation during initial orthodontic tooth movement in rats. Acta Histochem.

[37] Lausch E, Keppler R, Hilbert K, Cormier-Daire V, Nikkel S, Nishimura G (2009). Mutations in MMP9 and MMP13 determine the mode of inheritance and the clinical spectrum of metaphyseal anadysplasia. Am J Hum Genet.

[38] Austin JS, Gordon GM, Fini ME (2005). Global expression analysis of the MMP–TIMP gene families in corneal epithelial regeneration. Investig Ophthalmol Vis Sci.

[39] Olivieri J, Smaldone S, Ramirez F (2010). Fibrillin assemblies: extracellular determinants of tissue formation and fibrosis. Fibrogenesis Tissue Repair.

[40] Hubmacher D, Wang LW, Mecham RP, Reinhardt DP, Apte SS (2015). Adamtsl2 deletion results in bronchial fibrillin microfibril accumulation and bronchial epithelial dysplasia—a novel mouse model providing insights into geleophysic dysplasia. Dis Model Mech.

[41] Zhou C, Yang G, Chen M, Wang C, He L, Xiang L (2015). Lhx8 mediated Wnt and TGFbeta pathways in tooth development and regeneration. Biomaterials.

[42] Chen J, Liao L, Lan T, Zhang Z, Guo W (2020). Treated dentin matrix-based scaffolds carrying TGF-β1/BMP4 for functional bio-root regeneration. Appl Mater Today.

[43] Mill C, Monk BA, Williams H, Simmonds SJ, Jeremy JY, Johnson JL (2014). Wnt5a-induced Wnt1-inducible secreted protein-1 suppresses vascular smooth muscle cell apoptosis induced by oxidative stress. Arterioscler Thromb Vasc Biol.

[44] Yuan J, Wang X, Ma D, Gao H, Zheng D, Zhang J (2020). Resveratrol rescues TNFalphainduced inhibition of osteogenesis in human periodontal ligament stem cells via the ERK1/2 pathway. Mol Med Rep.

[45] Zhang X, Chen Q, Liu J, Fan C, Wei Q, Chen Z (2017). Parthenolide promotes differentiation of osteoblasts through the Wnt/beta-catenin signaling pathway in inflammatory environments. J Interferon Cytokine Res.

[46] Liu X, Tan GR, Yu M, Cai X, Zhou Y, Ding H (2016). The effect of tumour necrosis factor-alpha on periodontal ligament stem cell differentiation and the related signaling pathways. Curr Stem Cell Res Ther.

[47] Kangari P, Talaei-Khozani T, Razeghian-Jahromi I, Razmkhah M (2020). Mesenchymal stem cells: amazing remedies for bone and cartilage defects. Stem Cell Res Ther.

