# Supplementary figures and images for: Residual periodontal ligament in the extraction socket promotes the dentin regeneration potential of DPSCs in the rabbit jaw
Source: Stem Cell Res Ther. 2023 Mar 20;14:47. doi: 10.1186/s13287-023-03283-x (PMC10029302; doi:10.1186/s13287-023-03283-x)

DPSCs\_PDLSCs--vs--DPSCs(Up): Gene Ontology Classification

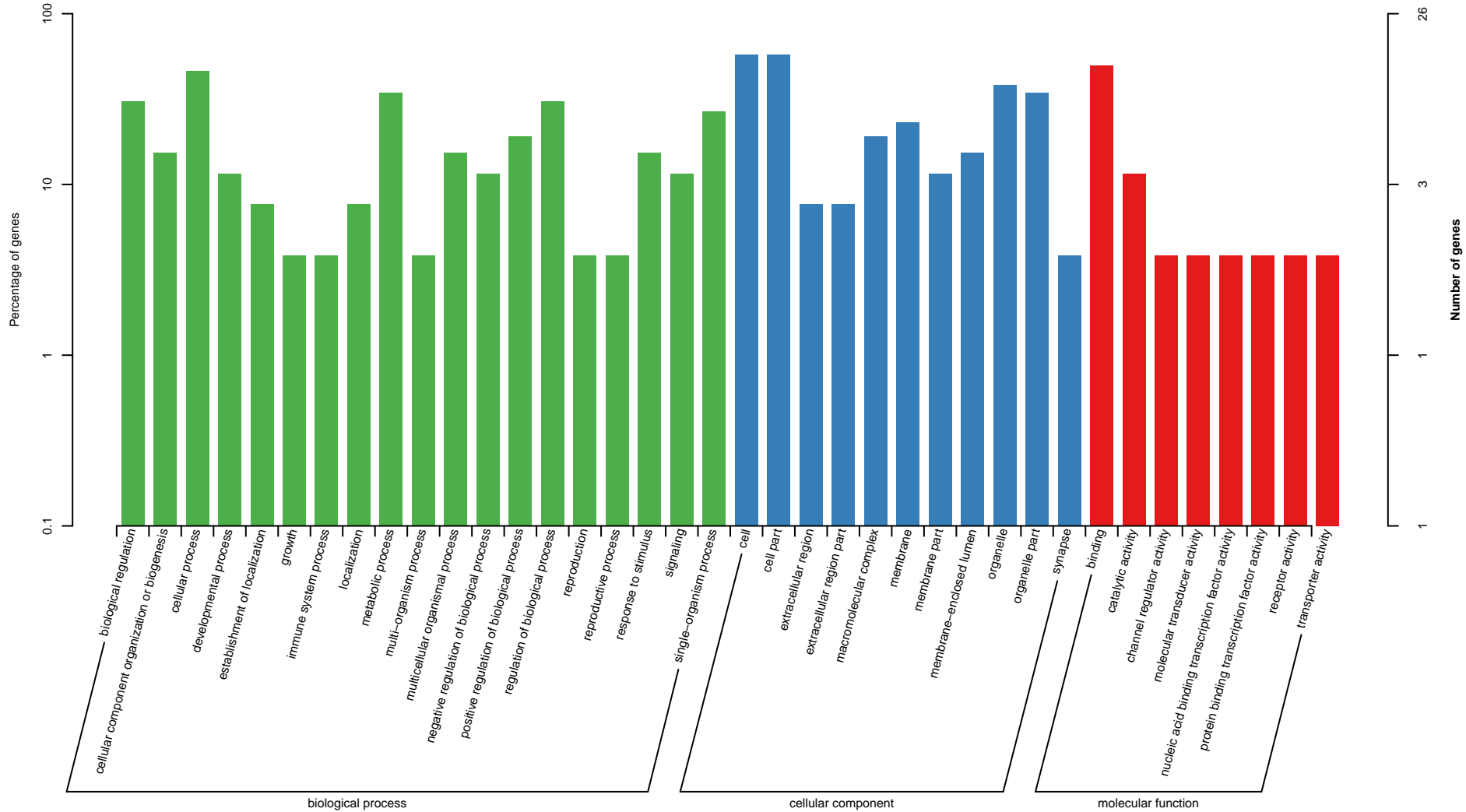

Supplement: Supplementary file 8 — Additional file 8: Upregulated GO functions of differentially expressed lncRNAs in DPSCs regulated by PDLSCs. [file 13287_2023_3283_MOESM8_ESM.pdf]

DPSCs\_PDLSCs--vs--DPSCs(Down): Gene Ontology Classification

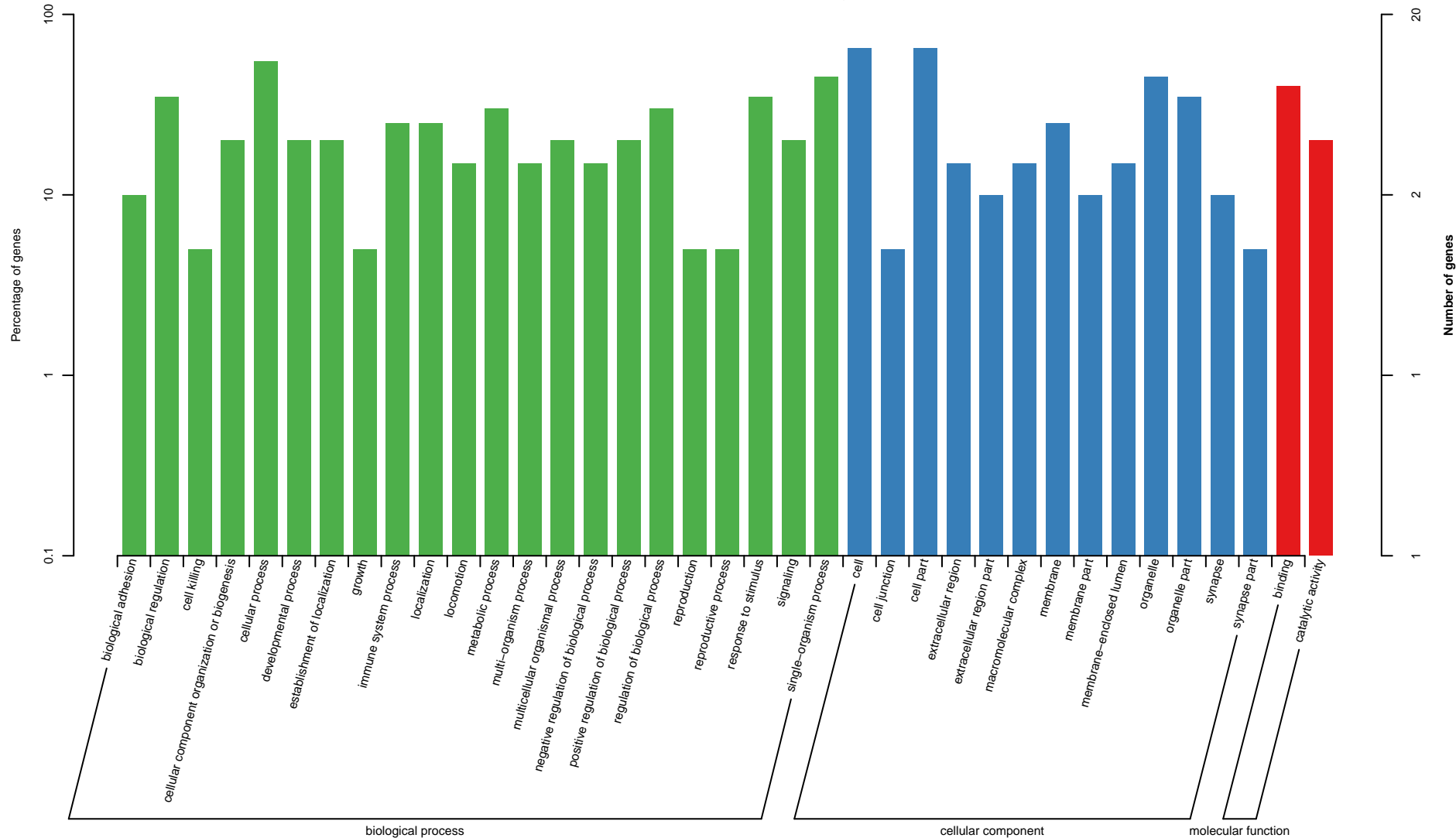

Supplement: Supplementary file 9 — Additional file 9: Downregulated GO functions of differentially expressed lncRNAs in DPSCs regulated by PDLSCs. [file 13287_2023_3283_MOESM9_ESM.pdf]

DPSCs\_PDLSCs-vs-DPSCs(Down): Top 30 GO Term

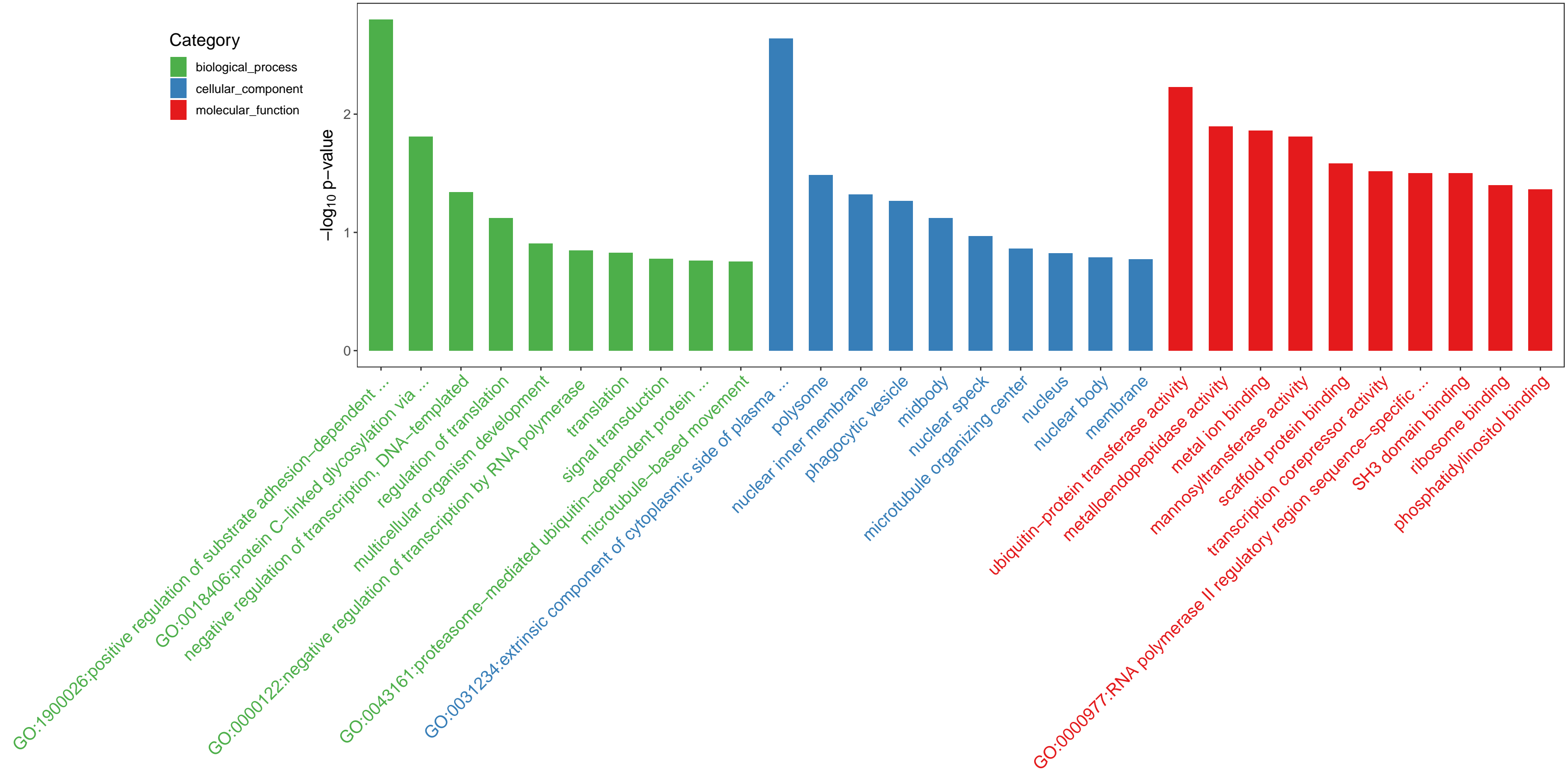

Supplement: Supplementary file 12 — Additional file 12: Downregulated GO functions of differentially expressed circRNAs in DPSCs regulated by PDLSCs. [file 13287_2023_3283_MOESM12_ESM.pdf]

DPSCs\_PDLSCs-vs-DPSCs(Total): Top 30 GO Term

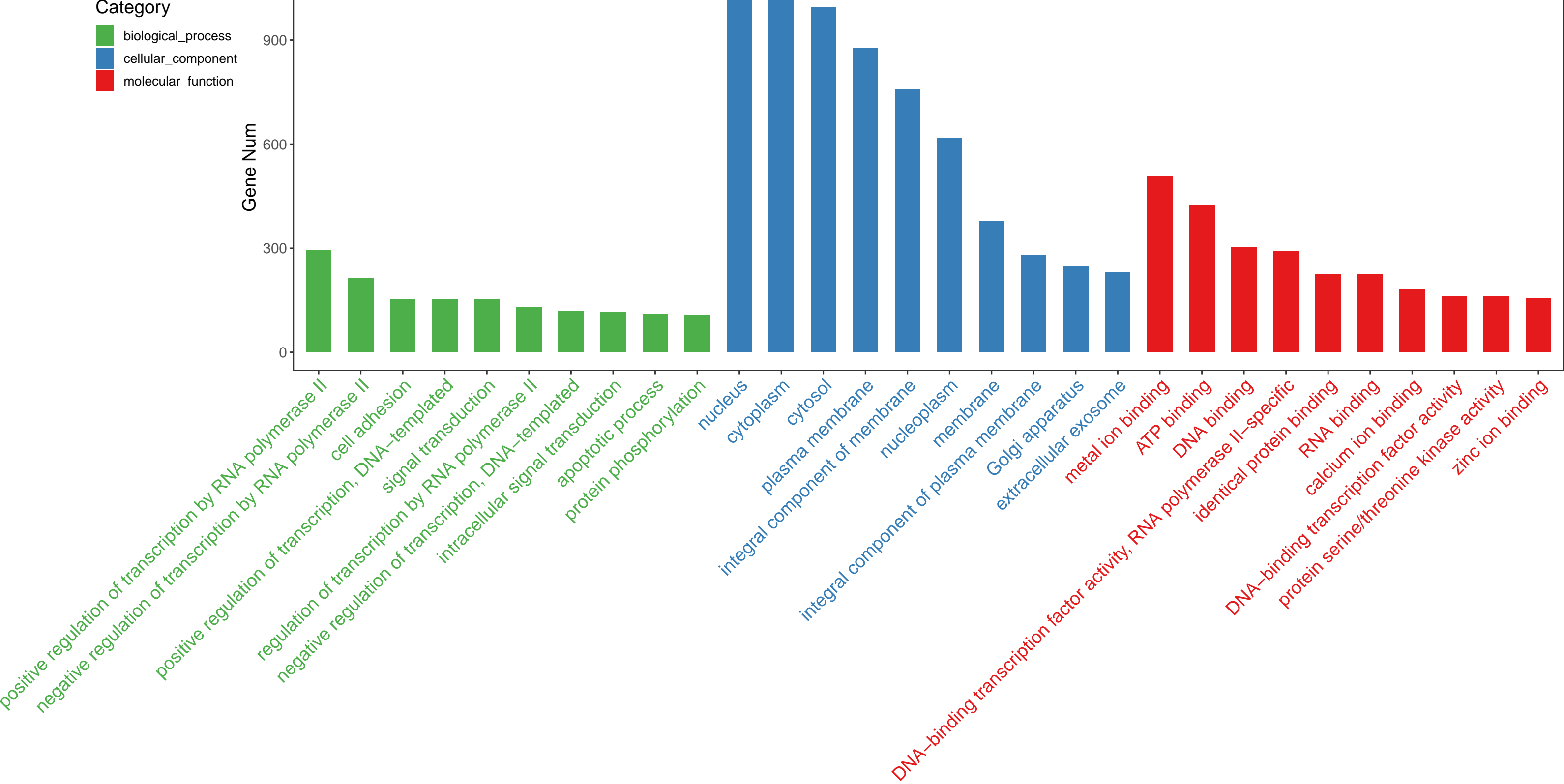

Supplement: Supplementary file 14 — Additional file 14: GO functions of differentially expressed miRNA target mRNAs in DPSCs regulated by PDLSCs. [file 13287_2023_3283_MOESM14_ESM.pdf]

# Top 20 of Enrichment

p-value

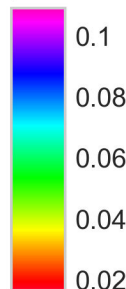

ListHit

1

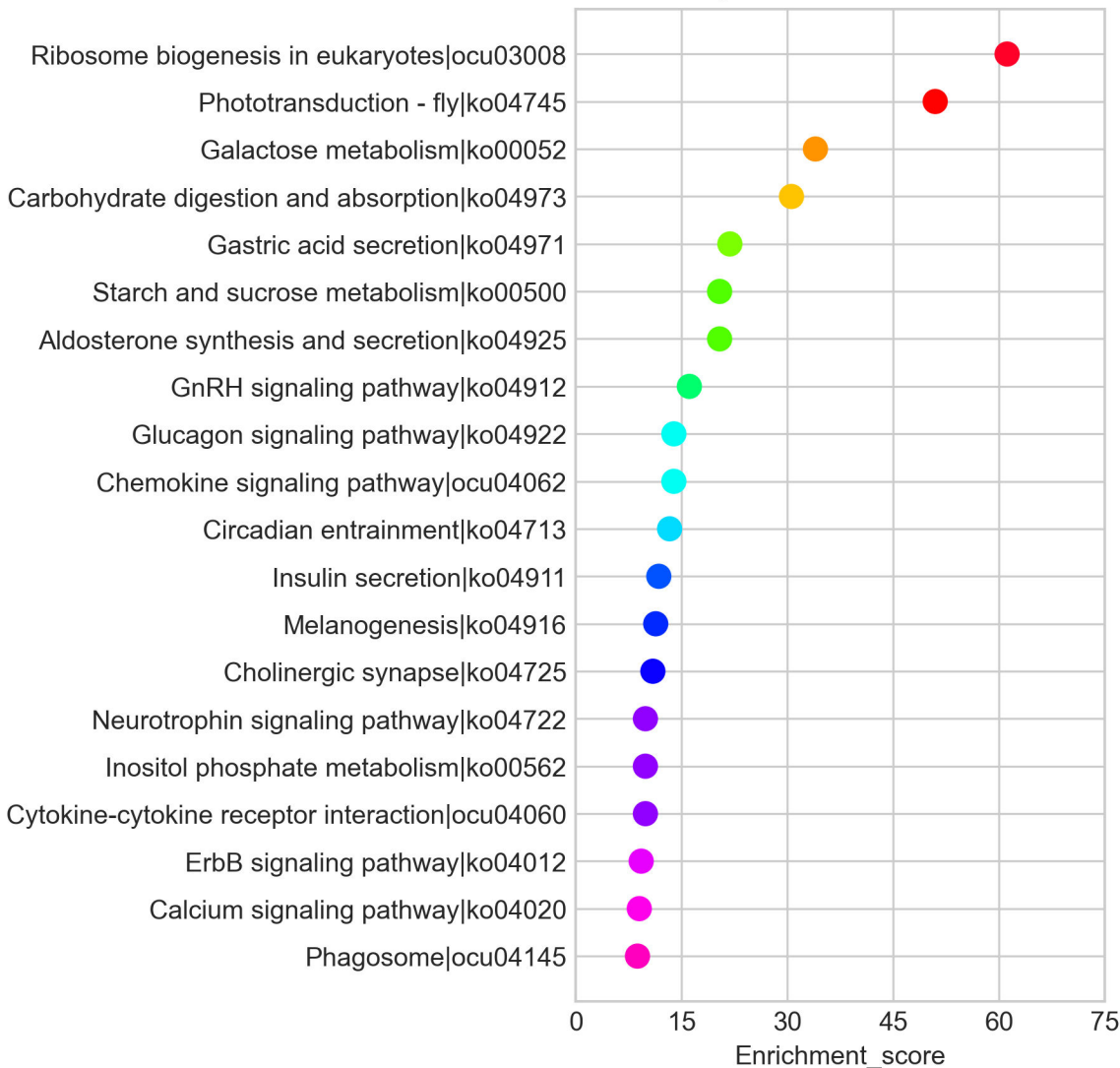

Supplement: Supplementary file 17 — Additional file 17: Upregulated pathways of differentially expressed lncRNAs in DPSCs regulated by PDLSCs. [file 13287_2023_3283_MOESM17_ESM.pdf]

# Top 20 of Enrichment

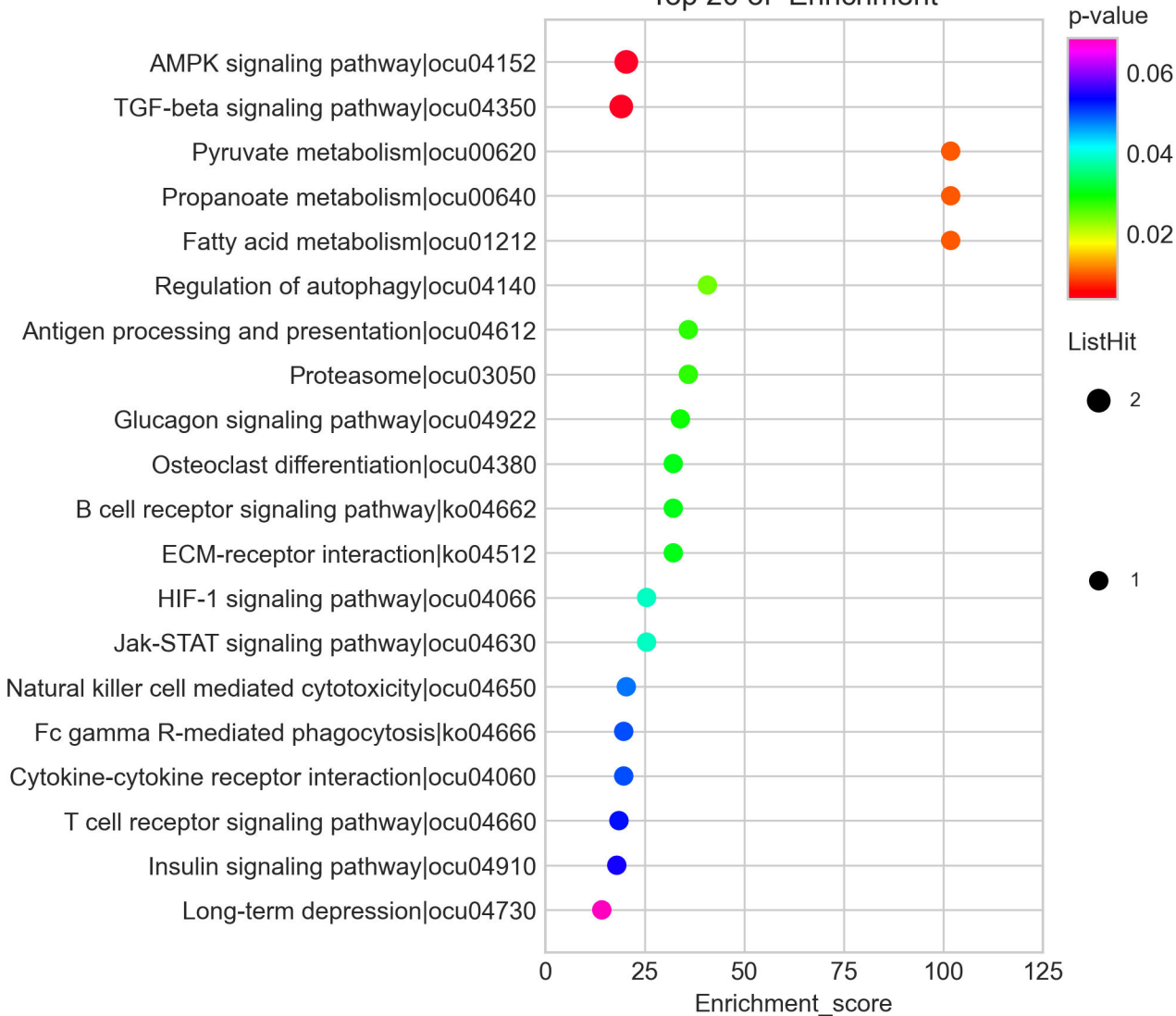

Supplement: Supplementary file 18 — Additional file 18: Downregulated pathways of differentially expressed lncRNAs in DPSCs regulated by PDLSCs. [file 13287_2023_3283_MOESM18_ESM.pdf]

# DPSCs\_PDLSCs-vs-DPSCs(Up): KEGG Enrichment top 20

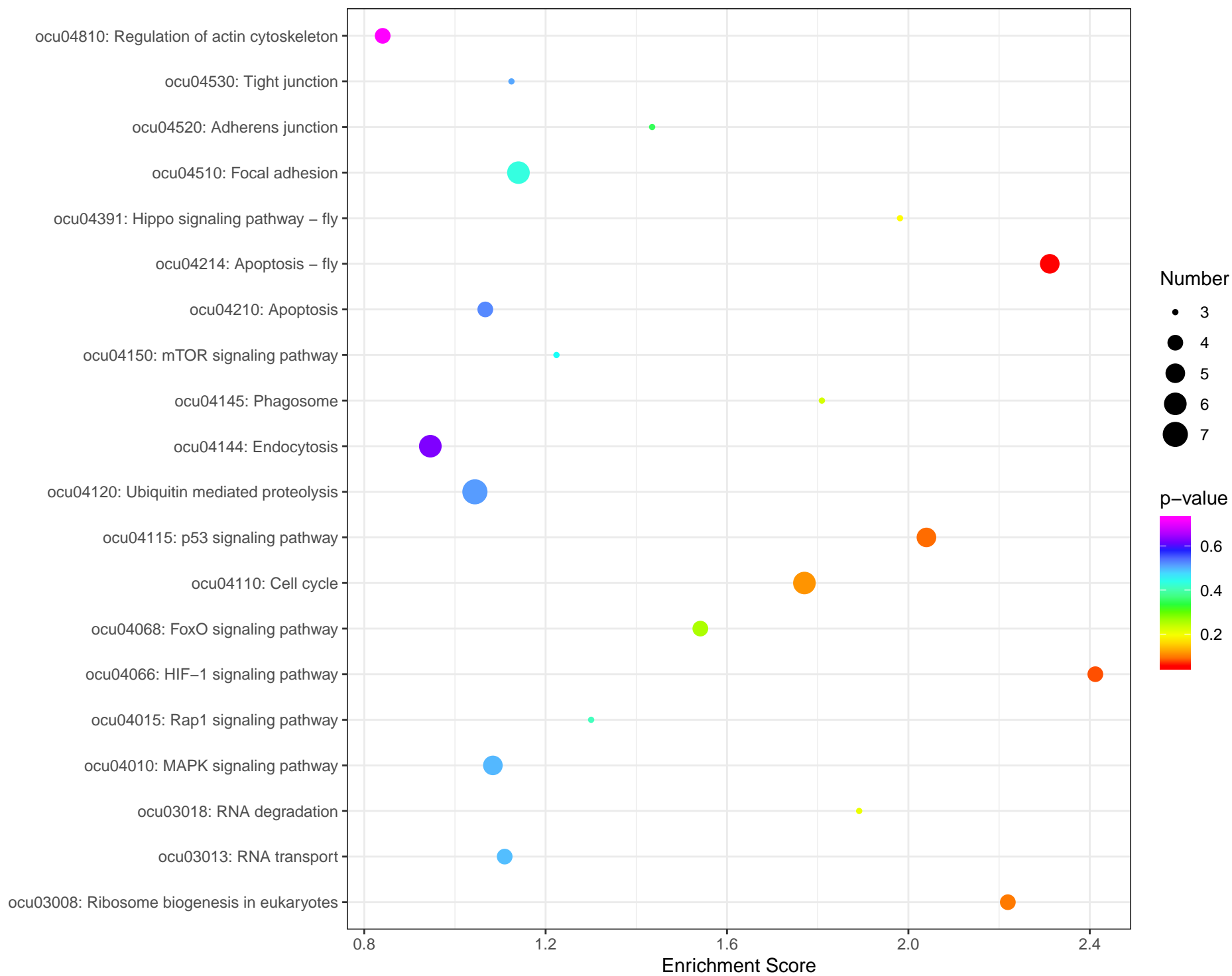

Supplement: Supplementary file 20 — Additional file 20: Upregulated pathways of differentially expressed circRNAs in DPSCs regulated by PDLSCs. [file 13287_2023_3283_MOESM20_ESM.pdf]

# DPSCs\_PDLSCs-vs-DPSCs(Down): KEGG Enrichment top 20

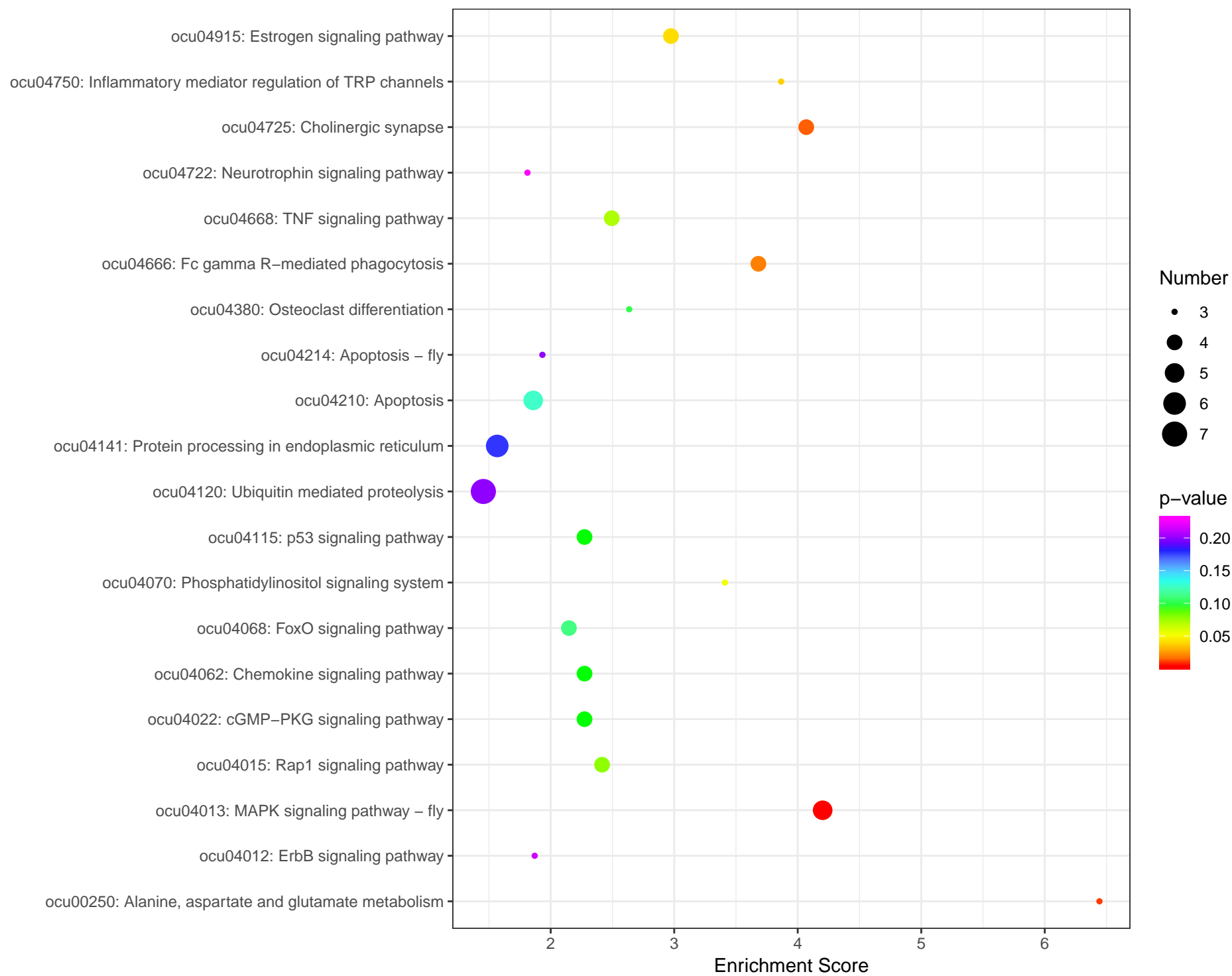

Supplement: Supplementary file 21 — Additional file 21: Downregulated pathways of differentially expressed circRNAs in DPSCs regulated by PDLSCs. [file 13287_2023_3283_MOESM21_ESM.pdf]

# DPSCs\_PDLSCs-vs-DPSCs(Total): KEGG Enrichment top 20

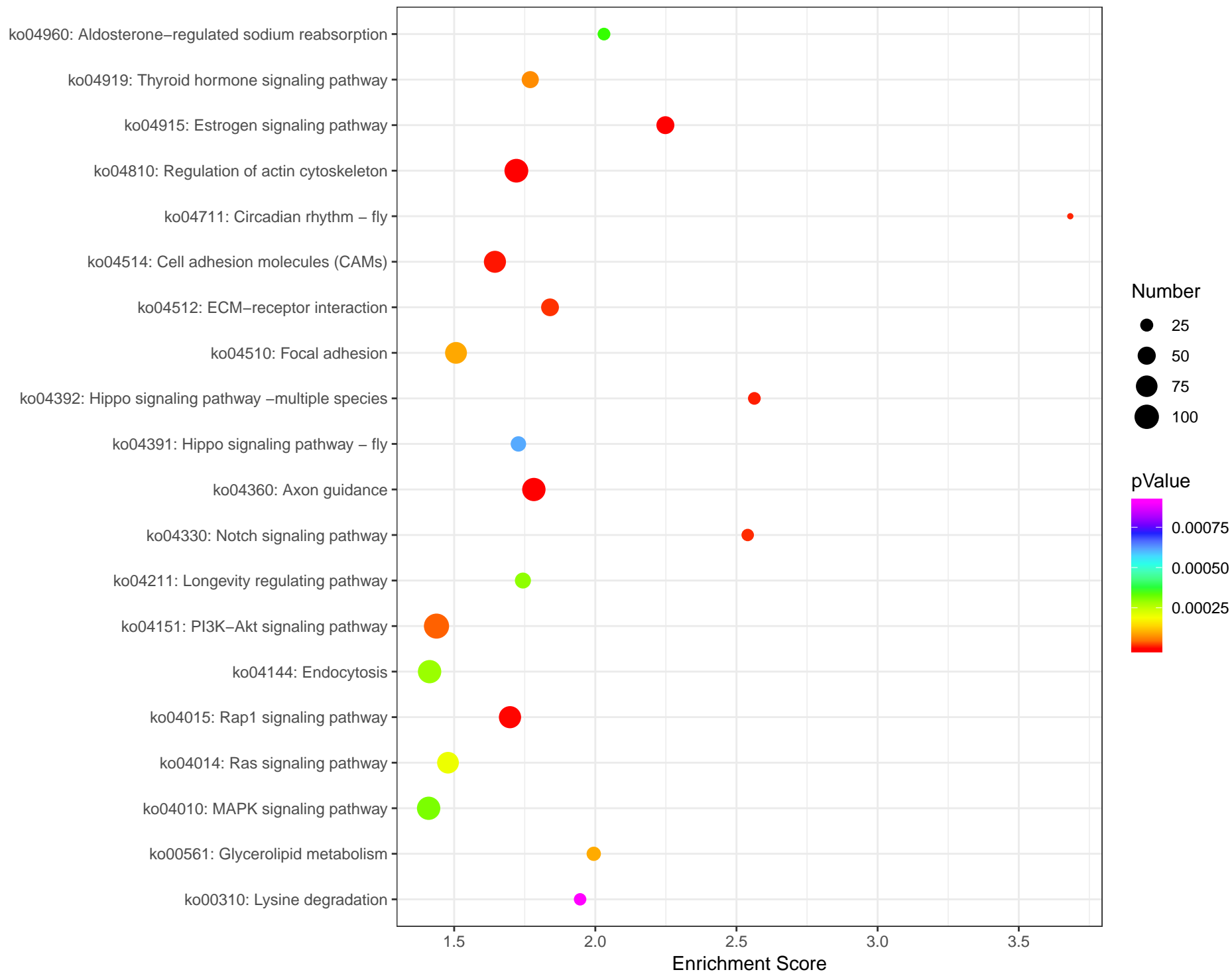

Supplement: Supplementary file 23 — Additional file 23: Pathways of differentially expressed miRNA target mRNAs in DPSCs regulated by PDLSCs. [file 13287_2023_3283_MOESM23_ESM.pdf]
