# Supplementary material for: Residual periodontal ligament in the extraction socket promotes the dentin regeneration potential of DPSCs in the rabbit jaw
Source: Stem Cell Res Ther. 2023 Mar 20;14:47. doi: 10.1186/s13287-023-03283-x (PMC10029302; doi:10.1186/s13287-023-03283-x)
Supplement: Supplementary file 11 — Additional file 11: Upregulated GO functions of differentially expressed circRNAs in DPSCs regulated by PDLSCs. [file 13287_2023_3283_MOESM11_ESM.pdf]

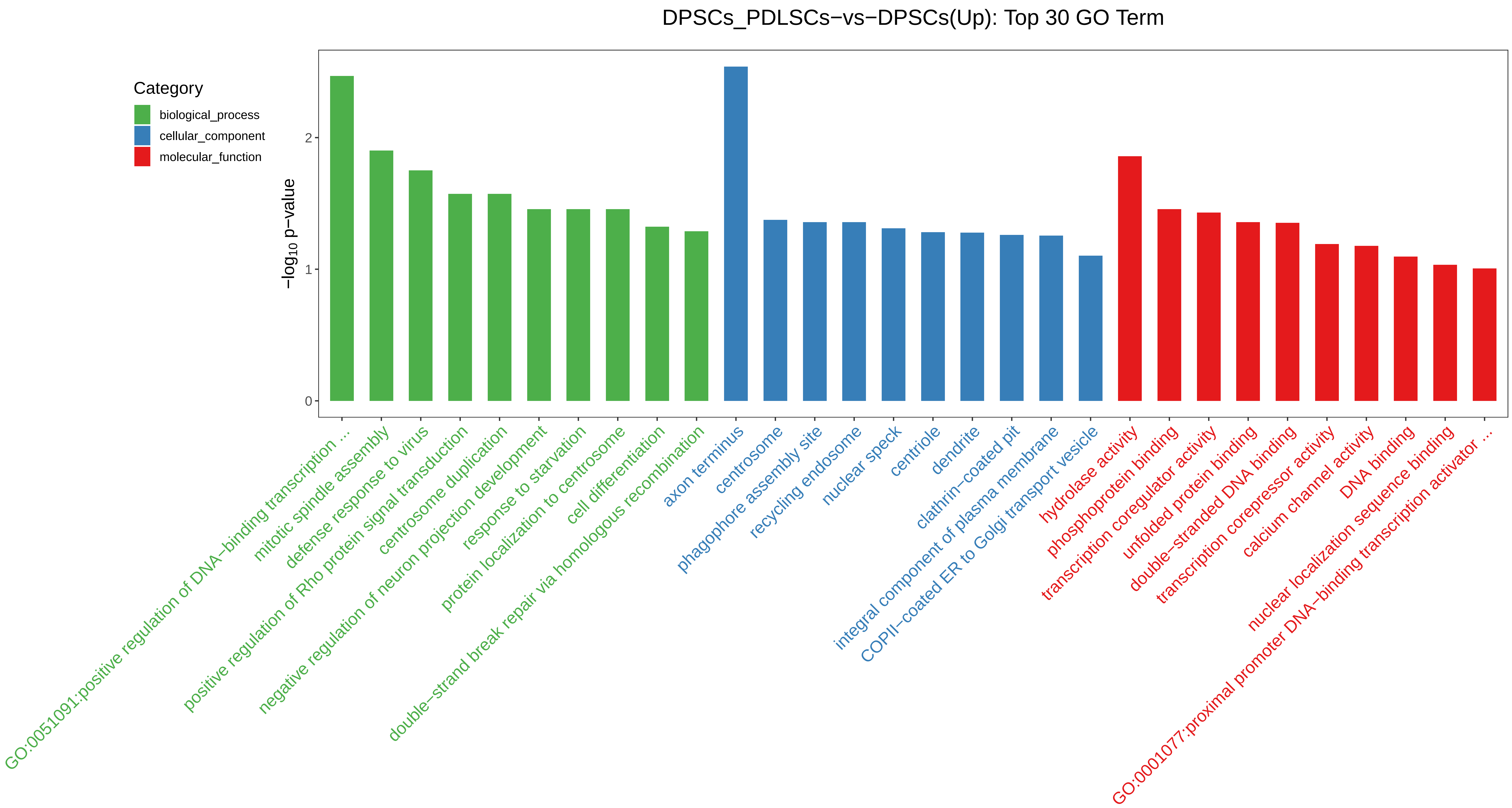
DPSCs_PDLSCs−vs−DPSCs(Up): Top 30 GO Term
Category
biological_process
cellular_component
molecular_function
−log10 p−value
0
1
2
GO:0051091:positive regulation of DNA−binding transcription ...
mitotic spindle assembly
defense response to virus
positive regulation of Rho protein signal transduction
centrosome duplication
negative regulation of neuron projection development
response to starvation
protein localization to centrosome
cell differentiation
double−strand break repair via homologous recombination
axon terminus
centrosome
phagophore assembly site
recycling endosome
nuclear speck
centriole
dendrite
clathrin−coated pit
integral component of plasma membrane
COPII−coated ER to Golgi transport vesicle
hydrolase activity
phosphoprotein binding
transcription coregulator activity
unfolded protein binding
double−stranded DNA binding
transcription corepressor activity
calcium channel activity
DNA binding
nuclear localization sequence binding
GO:0001077:proximal promoter DNA−binding transcription activator ...
